# Nanoparticles Surface Chemistry Influence on Protein Corona Composition and Inflammatory Responses

**DOI:** 10.3390/nano12040682

**Published:** 2022-02-18

**Authors:** Laura E. González-García, Melanie N. MacGregor, Rahul M. Visalakshan, Artur Lazarian, Alex A. Cavallaro, Svenja Morsbach, Agnieszka Mierczynska-Vasilev, Volker Mailänder, Katharina Landfester, Krasimir Vasilev

**Affiliations:** 1 UniSA STEM, Future Industries Institute, University of South Australia, Adelaide, SA 5095, Australia; laura.gonzalez_garcia@mymail.unisa.edu.au (L.E.G.-G.); rahul.madathiparambil_visalakshan@mymail.unisa.edu.au (R.M.V.); alex-anthony.cavallaro@unisa.edu.au (A.A.C.); 2 Flinders Institute for Nanoscale Science & Technology, College of Science and Engineering, Flinders University, Adelaide, SA 5042, Australia; 3 Max Planck Institute for Polymer Research, Ackermannweg 10, 55128 Mainz, Germany; lazarian@mpip-mainz.mpg.de (A.L.); morsbachs@mpip-mainz.mpg.de (S.M.); mailaend@mpip-mainz.mpg.de (V.M.); landfest@mpip-mainz.mpg.de (K.L.); 4 The Australian Wine Research Institute, Waite Precinct, Hartley Grove cnr Paratoo Road, Adelaide, SA 5064, Australia; agnieszka.mierczynska@awri.com.au; 5 Department of Dermatology, University Medical Center of the Johannes Gutenberg-University Mainz, Langenbeckstr. 1, 55131 Mainz, Germany; 6 College of Medicine and Public Health, Flinders University, Sturt Road, Adelaide, SA 5042, Australia

**Keywords:** plasma polymerization, silica nanoparticles, protein corona, inflammatory responses

## Abstract

Nanoparticles are widely used for biomedical applications such as vaccine, drug delivery, diagnostics, and therapeutics. This study aims to reveal the influence of nanoparticle surface functionalization on protein corona formation from blood serum and plasma and the subsequent effects on the innate immune cellular responses. To achieve this goal, the surface chemistry of silica nanoparticles of 20 nm diameter was tailored via plasma polymerization with amine, carboxylic acid, oxazolines, and alkane functionalities. The results of this study show significant surface chemistry-induced differences in protein corona composition, which reflect in the subsequent inflammatory consequences. Nanoparticles rich with carboxylic acid surface functionalities increased the production of pro-inflammatory cytokines in response to higher level of complement proteins and decreased the number of lipoproteins found in their protein coronas. On another hand, amine rich coatings led to increased expressions of anti-inflammatory markers such as arginase. The findings demonstrate the potential to direct physiological responses to nanomaterials via tailoring their surface chemical composition.

## 1. Introduction

Nanoparticles are widely used in biomedical applications for imaging, diagnosis and as carriers for therapeutic agents [1,2]. In the field of vaccine delivery, nanocarriers present several advantages over traditional vaccines. Specifically, nanocarrier encapsulation increases antigen stability and prevents its degradation. They enhance antigen recognition and can increase cellular uptake. They also prolonged the exposure of the antigen to the immune system by inducing antigen depot formation. Furthermore, nanocarriers can be functionalized to target specific immune cell receptors and lymphoid organs, improving adjuvant and antigen delivery towards a selective immune response [3,4,5,6]. In all cases, the fate of the treatment will be determined by the interactions of the nanomaterial with biological fluid and the subsequent immune cell responses. Influencing factors include nanoparticle size, surface area, surface chemical functionalities, curvature and dispersibility [7,8].

The fate of nanoparticles following administration into living systems is governed by the nature of the proteins adsorbing to their surface [9,10,11]. When nanoparticles come in contact with biological fluids, proteins and biomolecules rapidly adsorb on their surface, forming a protein shell [12]. This protein layer, called protein corona, confers a new identity to the nanoparticles modifying their physicochemical properties and, therefore, altering their pharmacological profile [13]. The formation and nature of the protein corona depends on the biological environment (i.e., serum, blood, plasma) as well as the physicochemical properties of the nanomaterials. SiO_2_ nanoparticles incubated with plasma can adsorb proteins in higher concentration, which may cause a stronger immune response than those incubated with serum since plasma contains a higher concentration of opsonin proteins than serum [14,15]. It is also known that hydrophobic nanoparticles or those containing greater surface charge attract more proteins [16]. Likewise, the subsequent interaction with biological cells depends on surface chemistry. For instance, nanoparticles with positive surface charge have often been associated with an increased cellular uptake when compared to negatively charged nanoparticles [17,18]. The modulation of nanoparticle surface charge also significantly influences the immune response. It is frequently recognized that positively charged nanoparticles activate the inflammatory response of immune cells, while neutral or negatively charged nanoparticles cause significantly less inflammation [19,20,21]. The protein corona bound to nanoparticles can either enhance or diminish the immune response. For example, Yan et al. demonstrated the contrasting roles of the protein corona formed on poly(methacrylic acid) nano-porous particles and the innate immunity [22]. Their results shown that the presence of the protein corona on the nanoparticles boosts the cellular uptake by macrophages, while the opposite effect was seen for monocytes. It is now well accepted that the nanoparticle materials, size, curvature, charge, and adsorbed surfactants all influence the protein adsorption and subsequent immunological responses. However, a comprehensive understanding of the unique influence of surface chemistry, without the interference of surfactants, on protein corona formation while nanoparticle size, shape and curvature remain identical is still lacking. 

Herein, we address these outstanding questions by tailoring in a controlled manner the surface chemical composition of silica nanoparticles and evaluate how the surface modification affects protein corona formation and innate immune cellular responses. We selected silica nanoparticles since these nanomaterials have attracted significant interest in a number of clinical applications [23,24,25]. They are also biocompatible and can be inexpensively and controllably produced in different sizes and shapes [26]. Silica nanoparticle surface functionality can be modified by the well-known silane chemistry that enables various biomolecules and other ligands to bind [27]. However, these wet processes are tedious, require multiple steps and are prone to contamination by silanes and by-product of the reactions [28]. A potent alternative to the classic surface modification routes is plasma polymerization, a technique that uses the plasma state of a purposely selected precursor to form a very thin polymeric film with desired properties. Although plasma polymers are traditionally used for coating of planar surfaces, recent reports demonstrated the capacity for efficient surface modification of micro and nanoparticles [29,30,31]. The careful selection of precursor structure allows for the deposition of coatings that exhibit a range of chemical functions including amines [32], acids [33], oxazolines [34], aldehydes [35], fluorocarbons [36] and many others. Other major benefits of the technology is that no solvents are involved and coatings can be deposited on practically any type of material without previous modification [37]. In comparison, other surface modification techniques require the use of surface-active agents such as surfactants, which influence the protein corona adsorption, or are limited to selected surfaces, e.g., silanization requires hydroxyl group functional surfaces and thiols require noble metal surfaces [38].

The experimental work presented here provides one of the first investigations on the protein corona formation and the effect of the innate immune response in a novel type of surface functionalized nanoparticles. In this work, we used plasma polymerization to finely tailor the outermost surface chemistry on silica nanoparticles (SiNPs). These plasma polymer-coated silica nanoparticles (PPSiNPs) with distinct chemical functional groups i.e., amine, carboxylic acid, oxazolines, and alkanes were incubated in human serum and plasma. The resulting protein coronas were characterized using SDS-PAGE and LC-MS to reveal their composition. Finally, the influence of the protein coronas on the innate immune response was evaluated in culture of human macrophages.

## 2. Materials and Methods

### 2.1. Nanoparticle Design and Characterization

#### 2.1.1. Functionalization of Silica Nanoparticles

Silicon dioxide nanoparticles (10–20 nm) were purchased from Sigma Aldrich (St. Louis, MO, USA) and functionalized. Plasma polymerization was carried out in a custom-built plasma reactor equipped with an agitation platform specially designed for coating powder materials as described previously [30,39]. Briefly, the organic precursor (2-methyl-2-oxazoline, allylamine, acrylic acid or 1,7-octadiene) was introduced in the vacuum chamber at 0.23 mbar pressure and polymerized at a power of 10 W for acrylic acid, 40 W for allylamine and 50 W for 2-methyl-2-oxazoline and 1,7-octadiene. The deposition time was 10 min for all the monomers as optimized on our previous work [29,40].

#### 2.1.2. X-ray Photoelectron Spectroscopy (XPS)

Surface chemical composition of plasma polymerized silica nanoparticles was analyzed using a Kratos AXIS Ultra DLD spectrometer (Manchester, UK). XPS spectra were recorded with a monochromatic AlKα (hν = 1486.7 eV) radiation source conducted at an electric current of 15 mA and a voltage of 15 KeV. Survey spectra was recorded over a range of 0–1100 eV at a pass energy of 160 eV and high-resolution spectra was performed at a pass energy of 20 eV. Casa XPS software was used for data analysis. All binding energies were referenced to the aliphatic carbon C1s peak at 285.0 eV. Curve fitting of core level envelopes was performed with the minimum number of Gaussian–Lorentzian components, normally at 30% Lorenzian and 70% Gaussian functions.

#### 2.1.3. Time of Flight Secondary Ion Mass Spectrometry (ToF-SIMS)

ToF-SIMS was performed to characterize the chemistry of the plasma-coated particles using a PHI TRIFT V nanoTOF instrument (Physical Electronics Inc., Chanhassen, MN, USA) equipped with a pulsed liquid metal Au primary ion gun (LMIG) operated at 30 kV in a bunched Au+ mode to optimize mass resolution. All spectra were analyzed using WincadenceN1 software (Ver. 1.8.1.3 Physical Electronics Inc.) and further studied using NESAC/BIO ToolBox developed at the University of Washington (Seattle, WA, USA) by Dan Graham.

#### 2.1.4. Zeta-Potential

The dynamic electrophoretic mobility of bare and plasma polymer-coated silica nanoparticles was measured on a Malvern Zetasizer Nano ZS and the zeta potential was calculated by applying the Smoluchowsky equation [41]. Measurements were carried out in 10^−3^ M NaCl solution at 25 °C in a pH range from 1 to 11.

#### 2.1.5. Fluorescence Imaging

Autofluorescence from the plasma polymer coatings was imaged in an Olympus IX83 Fluorescence Microscope with Cool LED (Tokyo, Japan). Bulk powder samples were placed in a 96 well plate and imaged at 10× magnification both with bright field and 490 nm excitation filters over a 0.32 cm^2^ area.

#### 2.1.6. Transmission Electron Microscopy

SiNPs and PPSiNPs size and shape distributions were characterized using a high resolution transmission electron microscope JEOL-2100F (JEOL, Tokyo, Japan). TEM samples were prepared by drying a droplet of a SiNPs suspension on a carbon-coated copper grid (ProSciTech, Kirwan, Australia). The images were processed using image J software and particle size distribution was analyzed. 

### 2.2. Protein Corona Study

#### 2.2.1. Protein Corona Preparation

Pooled human blood plasma and serum (heparin anticoagulant) were purchased from Innovative Research and stored at −80 °C. In order to remove any aggregated proteins plasma and serum samples were span down at 4 °C, 20,000× *g* for 1 h before use. Plasma polymer-coated silica nanoparticles were dispersed in ultrapure water in a 1% *w/v* concentration by probe ultrasonication for 2 min at 120 μm amplitude. 100 µL of this dispersion was incubated with 1 mL plasma or serum at 37 °C for 1 h under constant agitation. The samples were then centrifuged at 20,000× *g* for 1 h to separate the particles from the supernatant. Soft corona proteins were eliminated by resuspension of the particles in 1 mL PBS followed by centrifugation at 4 °C, 20,000× *g* for 1 h, three times [11]. In the last step, the centrifugation pellet was dispersed in 150 µL of 62.5 mM Tris-HCL supplemented with 2 wt% SDS and incubated for 15 min at 90 °C to desorb the proteins constituting the hard corona [42]. The amounts of surface bound proteins eluted from the plasma polymer-coated silica nanoparticles in this last step were determined by Pierce 660 nm protein assay (Thermo Fisher Scientific, Waltham, MA, USA) following the manufacturer instructions.

#### 2.2.2. SDS Polyacrylamide Gel Electrophoresis (SDS-PAGE)

For SDS-PAGE, 1 µg of the hard protein corona samples were mixed with LDS reducing agent and sample buffer and incubated for 10 min at 70 °C. The samples were then applied on a NuPAGE 10% Bis-Tris protein gel (Thermo Fisher Scientific) and run for 1 h at 100 mV. The protein bands were stained using a SilverQuest Silver Staining Kit (Thermo Fisher Scientific) following the manufacturer instructions.

#### 2.2.3. Digestion of the Protein Corona for Mass Spectrometry Analysis

SDS was removed from the samples by Pierce detergent removal columns (Thermo Fisher). Proteins were digested as previously described in details by Tenzer et al. [43]. Briefly, proteins were precipitated with ProteoExtract protein precipitation kit (CalBioChem, San Diego, CA, USA) and the resulting protein pellets were resuspended in 0.1% RapiGest SF (Waters Corporation, Milford, MA, USA) in 50 mM ammonium bicarbonate and incubated for 15 min at 80 °C. 5 mM dithiothreitol was added to reduce the proteins and incubated at 56 °C for 45 min. 15 mM Iodoacetamide was then added and incubated in the dark at room temperature for 1 h. Tryptic digestion with 50:1 ratio of protein:trypsin was carried out for 18 h at 37 °C. The reaction was quenched by the addition of 2 μL hydrochloric acid.

#### 2.2.4. Liquid Chromatography Mass Spectrometry (LC-MS) Analysis

LC-MS analysis was carried out in a nanoACQUITY ultra high-performance liquid chromatography (UPLC) system coupled with a Synapt G2-Si mass spectrometer (Waters Corporation). Digested peptides were injected into a C18 nanoACQUITY Trap Column (5 μm, 180 μm × 20 mm, Waters Corporation) and separated at a C18 analytical reversed-phase column (1.7 μm, 75 μm × 150 mm) with a mobile phase A consisting of 0.1% (*v*/*v*) formic acid in water and a mobile phase B consisting of acetonitrile with 0.1% (*v*/*v*) formic acid at a flow rate of 0.3 μL min^−1^, using a gradient of 2–40% mobile phase B for 70 min. 150 fmol μL^−1^ Glu-Fibrinopeptide was infused at a flow rate of 0.5 μL min^−1^ as a reference compound.

Electrospray Ionization was performed in positive ion mode with NanoLockSpray source and mass spectrometry was carried out in resolution mode. Data acquisition was operated in a mass to charge range of 50–2000 Da, scan time of 1 s from 20 to 40 V ramped trap collision energy 20 to 40 V and a total acquisition time of 90 min. Each sample was run in two technical replicates.

Data was acquired with MassLynx 4.1 and processed with Progenesis QI for Proteomics Version 2.0 software using a reviewed human data base (Uniprot). As noise reduction thresholds for low energy, high energy and peptide intensity were set to 120, 250, and 750 counts were used. The following criteria were used for analysis: fixed carbamidomethyl modifications for cysteine, variable oxidation for methionine, one missed cleavage, max. protein mass 600 kDa, and a false discovery rate of 4%. Protein identification required at least two assigned peptides and five assigned fragments; for peptide identification, two assigned fragments were needed. Quantitative identification of each identified protein in fmol was provided based on the TOP3/Hi3 approach. 

The results were plotted using Graphpad prism software and the data was shown as mean ± standard deviation.

### 2.3. Cell Viability

Human fibroblasts (HFFF2, Sigma Aldrich, St. Louis, MO, USA) were culture in Dulbecco’s Modified Eagle Medium DMEM (Life Technologies, Carlsbad, CA, USA) supplemented with 10% fetal bovine serum (FBS, Thermo Scientific) and 1% (*v/v*) penicillin/streptomycin (Life Technologies). The cell viability was assessed using the resazurin assay (Sigma Aldrich). HFFF2 cells were seeded on a 96 well plate at a density of 10^4^ cells per well. After overnight growth, the medium was removed, and the cells were washed with PBS. Fresh medium with SiNPs at concentrations of 37.5, 75, and 150 µg/mL was then added to the wells and incubated for 24 h. Cell culture media containing 11 µg /mL resazurin was then added to each well and incubated for 1 h. The fluorescence was measured in a microplate spectrophotometer at 544 nm excitation and 590 nm emission wavelengths. The percentage of cell viability was calculated as:

Cell viability %=(fluorescence intensity of treatedfluorescence intensity of control)×100

### 2.4. Immune Response Studies

Human monocytes (THP-1 cell line) were cultured in RPMI 1640 (Sigma Aldrich) media supplemented with 10%v fetal bovine serum (Thermo Scientific) and 1%v penicillin/streptomycin (Life Technologies) and maintained at 37 °C and 5% CO_2_ humidified atmosphere. Monocytes were differentiated into macrophages d-THP by treating them with 100 ng/mL phorbol 12-myristate 13-acetate (PMA, Sigma Aldrich) [44]. Differentiated dTHP-1 macrophages were seeded on 96 well plates at a density of 10^4^ cells per well. After overnight attachment the cells were washed with PBS and fresh serum-free media was added. The nanoparticles were then added to the wells at a concentration of 75 µg/mL along with 1 µg/mL lipopolysaccharides (LPS from E. coli O111:B4, Sigma Aldrich) to activate the dTHP-1 macrophages. After 6 h of incubation, conditioned media was collected and centrifuged at 20,000× *g* for 30 min to remove nanoparticles and cellular debris. Cytokines present in the conditioned media were analyzed using LEGENDplex ELISA kits (BioLegend, San Diego, CA, USA) following the manufacturer’s instructions.

## 3. Results and Discussion

### 3.1. Tuning Surface Chemistry of Silica Nanoparticles

**Figure 1:**
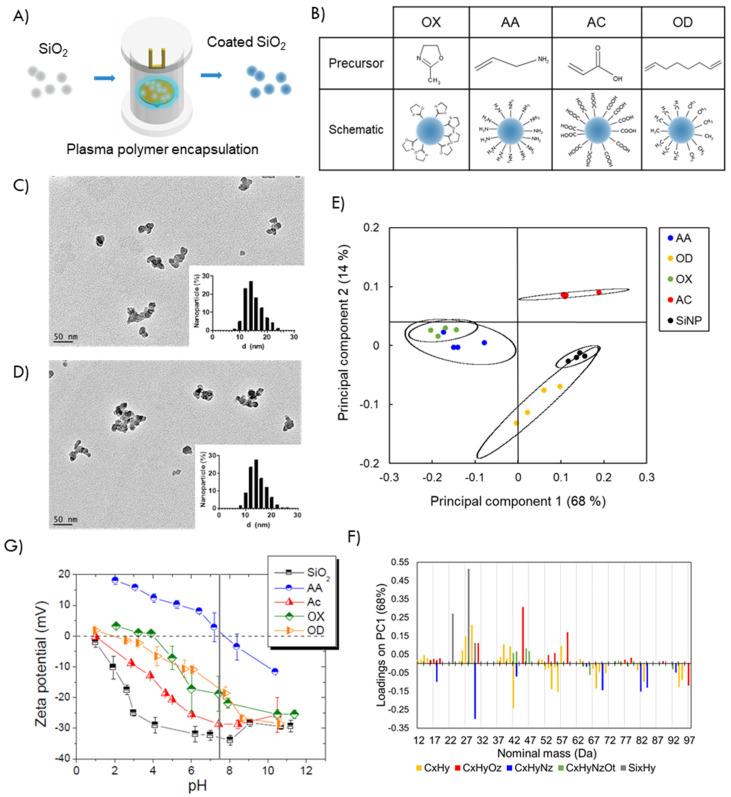
Characterization of PPSiNPs. (**A**) Schematic of the surface modification procedure of silica nanoparticles by plasma polymerization. (**B**) Chemical structure of the precursors used with representation of the expected surface functionalization. Methyl oxazoline (OX) provides SiNPs with heterocyclic functionality, Allylamine (AA) provides SiNPs with amine groups, Acrylic acid (AC) provides SiNPs with carboxyl acid groups, and 1,7-octadiene (OD) provides SiNPs with hydrocarbon coatings. (**C**) TEM image and size distribution histogram of uncoated SiNPs. (**D**) and after OD modification. (**E**) Principal component analysis of data obtained from ToF-SIMS characterization data. Scores plot of the first two principal components (PC1, 68% and PC2, 14%) for the surface modification of the SiNPs: bare SiO_2_ (black), AA (blue), AC (red), OD (yellow) and OX (green). (**F**) TOF-SIMS PC1 loadings for all fragments below 100 m/z. (**G**) Surface potential vs. pH of bare and plasma polymer-coated SiNPs measured in 10^−3^ M NaCl. Each point represents an average of three chemical repeats averaged by 3 technical repeats.

The surface chemistry of model silica nanoparticles (SiNPs) was modified using a solvent free, plasma-enabled thin film deposition (Figure 1A). The organic precursors were selected for their capacity to produce nano-thin plasma polymer films with specific chemical functionalities and surface charge. Figure 1B shows the chemical structures of the precursors used and a schematic representation of the surface architecture of the modified particles: acrylic acid (AC) provides films rich in carboxyl acid groups, allylamine (AA) tailors the particle surface with amine groups [45], methyl oxazoline (OX) gives heterocyclic functionality [46] and 1,7-octadiene (OD) provides pure hydrocarbon coatings [30]. These different plasma functionalities were selected to epitomize the chemical compositions of amino acids in proteins (-COOH, -NH_2_ and -CH_3_) and provide different surface charges [47].

Transmission electron microscopy showed that the SiNPs were amorphous in shape in agreement with the manufacturer description and the average size was 15.0 ± 3.2 nm. The plasma polymer layer was not visible and the average size of PPSiNPs was 14.8 ± 3.0 nm. Representative TEM images and size distribution histograms of bare SiNPs and plasma modified OD particles are shown in Figure 1C,D.

The chemical signatures of the PPSiNPs were analyzed by ToF-SIMS. The technique is extremely sensitive to the molecular structure of the first 1–2 nm of material surface. It detects low molecular weight fragment ions with high mass resolution. Principal component analysis (PCA) was conducted on the whole data set that comprised of the bare SiNPs, as well as the particles functionalized with the four distinct plasma deposited polymers i.e., AA, OX, AC and OD. PCA is a multivariate technique used to identify differences and similarities in ToF-SIMS spectra and classify spectra into groups. It reduces the multidimensionality of the mass spectra to ease interpretation. Principal component 1 (68%) was plotted against principal component 2 (14%). These first two principal component account for 82% of the total variance in the dataset. The results of PC1 versus PC2 is a bidimensional plane where the different nanoparticle coatings are well clustered in accurate representation of the original multidimensional matrix. (Figure 1E). PCA demonstrated distinct chemical differences between bare silica nanoparticles, OD, and AC coatings as their 95% confidence interval areas did not superimpose. OX and AA were clearly separated from OD, AC and bare SiNPs on PC2. However, possessing similar amine groups, OX and AA featured overlapping positive ion SIMS signals. Overall, the PCA analysis indicates that following plasma polymer coating, the nanoparticles displayed distinct outmost surface chemical signatures. The PC1 loadings plot (Figure 1F) shows the direction cosines between the PC1 axis and the mass spectra fragments of greatest variation. This detailed analysis of the loading fragments revealed that sample differences were attributed to 31 hydrocarbon fragment ions, 12 oxygen heteroatoms fragment ions, 9 nitrogen and oxygen containing fragment ions, 8 amine containing fragment ions and, lastly, 3 silane fragment ions. Individual comparisons of the plasma polymer coatings with the bare SiNPs (Figure S1) are further evidence of the distinct chemical variations of the deposited films, with prominent amine fragments on AA, oxygen heteroatoms on AC, nitrogen and oxygen fragments on OX and alkanes on OD.

Figure 1G shows the surface potential of the bare and PPSiNPs in the pH range from 1 to 11. As expected, in all samples studied, the surface potential decreases with increasing pH. The surface of bare SiNPs remain negatively charged over the pH range investigated with surface potential values varying from −2 to −33 mV. These results are in good agreement with those reported by others [48,49]. The increase of surface potential with decreasing values of pH can be attributed to the protonation of the particle surface oxygen to form Si-OH groups. AA is a strong basic compound that confers a positive surface potential value to AA-coated nanoparticles for pH lower than its isoelectric point of 7.7 [50]. OX also provides amine groups amongst other unique chemical functionalities [34,51], which overall confer the films a lower basic strength, corresponding to an isoelectric point of 4. The isoelectric point of alkyl-functionalized OD nanoparticles was at pH 2. AC coated particles had negative surface potential over the measured pH range due to the acidity of the carboxylic acid groups [50]. Most importantly, the surface modification provided PPSiNPs with a wide range of surface charges at physiological pH 7.4, vertical line in Figure 1G. Bare SiNPs were the most negatively charged at −32 mV followed by AC coatings at −29 mV. OX and OD had slightly negative charge at −18 mV and −16 mV, respectively, whereas AA coatings were positively charged at 2 mV, in good agreement with previous reports [52].

**Figure 2:**
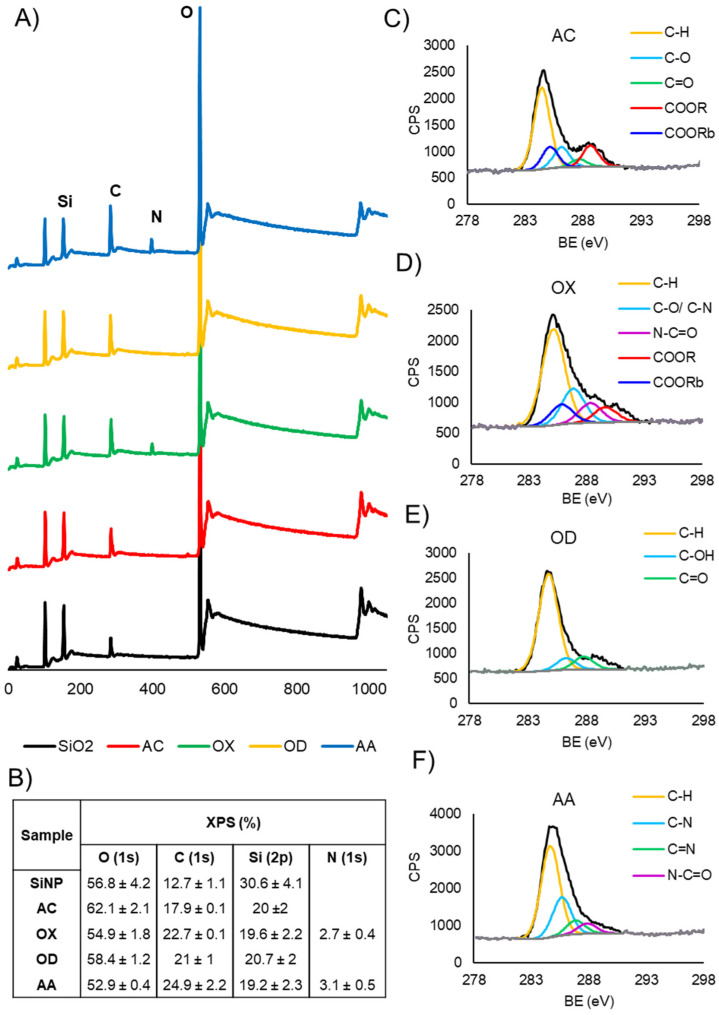
XPS analysis of PPSiNPs. (**A**) XPS survey spectra showing the surface chemical composition of bare and plasma polymer-coated silica nanoparticles. (**B**) Table of the atomic percentage of oxygen, carbon, nitrogen, and silica quantified from XPS analysis. High resolution C1s spectra obtained via XPS of (**C**) AC, (**D**) OX, (**E**) OD, and (**F**) AA.

The chemical elemental composition of the bare and plasma polymer-coated silica nanoparticles was analyzed by XPS. The survey spectra (Figure 2A) show that all samples contained carbon, oxygen and silica with AA and OX coated PPSiNPs featuring an additional nitrogen peak consistent with the chemical structure of these precursors. The table in Figure 2B provides a detailed elemental composition in atomic percentage obtained after quantification of the survey spectra. An important measure is the significant reduction in measurable silica content of the functionalized nanoparticles (19–20%) in comparison to the bare silica (30%), which is a direct consequence of the plasma polymer films covering the SiO_2_ surface. The presence of the Si(2p) signal in the coated nanoparticles can be explained by taking in consideration the sampling depth of XPS which is around 10 nm. Clearly, the plasma polymer films on the outer surface of the nanoparticles are thinner than 10 nm, thus, allowing the silicon signal to be detected. However, the strong change of the surface potential (Figure 1G) in accordance with the type of surface modification applied indicates that the nanoparticles are completely and uniformly coated by the relevant plasma polymer layer. The homogeneity of nanoparticles surface modification was further confirmed via autofluorescence analysis, as shown in Figure S2.

High resolution spectra of the C1s region (Figure 2C–F) provides further insights into the chemistry of the different plasma polymer films. All coatings contained a characteristic peak at 285.0 eV assigned to aliphatic hydrocarbons (C-C, C-H). A distinguishable O-C=O component at 289.2 eV (with a beta shift of this peak at 285.7 eV) in the spectrum of AC was assigned to carboxyl acid groups present in the coatings. A component at 288.2 eV assignable to amides (N-C=O) could be fitted in the OX C1s high resolution spectrum. OD contained a main component at 285.0 eV belonging to aliphatic hydrocarbons with smaller C-O and C=O components caused by partial oxidation, which is unavoidable upon exposure to air. Finally, the AA coating was characterized with components at 286.5 eV, 287.2 eV and 288.2 eV corresponding to amine groups (C-N), imine groups (C=N) and amide groups (N-C=O) respectively. The identified chemical compositions are consistent with those previously reported by us and others [34,51,53,54,55].

The chemical and physical analysis demonstrate that via plasma facilitated surface modification model nano-objects were successfully prepared. They have identical size but feature a range of surface chemistries and surface charges. These model nanoparticles were next used to study the influence of surface chemical properties without the influence of surfactants on protein binding from serum and plasma.

### 3.2. Influence of Different Plasma Polymer Chemistries on Protein Corona Formation

**Figure 3:**
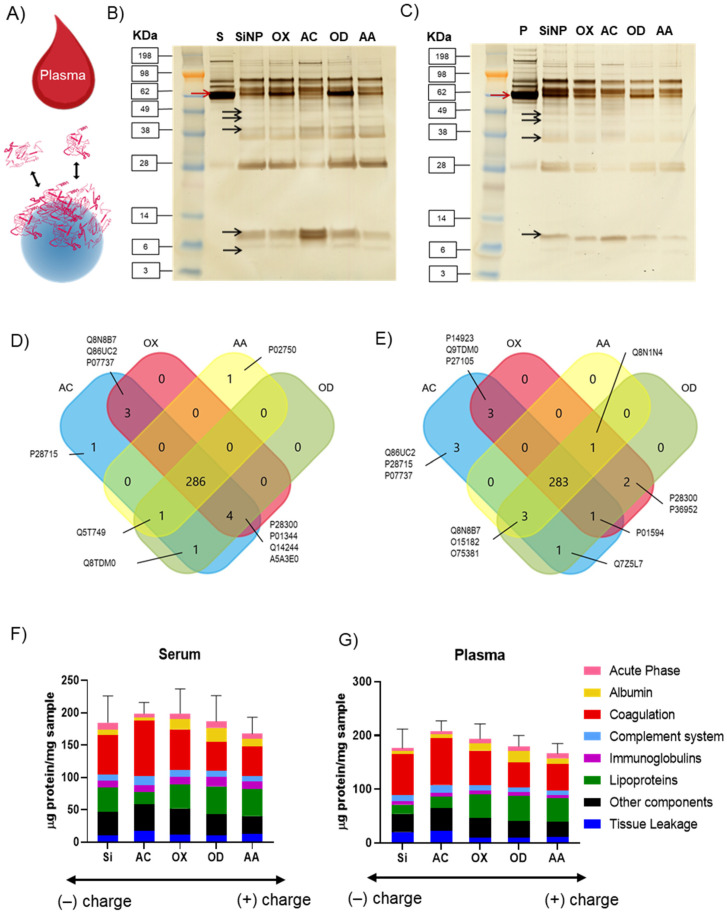
Protein corona formation on PPSiNPs. Schematic illustration of the blood-nanoparticle interactions and protein corona formation phenomena (**A**) SDS-PAGE of protein corona composition formed around silica nanoparticles with different plasma polymer surface modification in human plasma (**B**) and serum (**C**). Venn diagram showing the overlapping identified proteins (labelled by their accession numbers) in the serum (**D**) and plasma coronas (**E**). Quantification of human serum (**F**) and plasma (**G**) proteins adsorbed on SiNPs and PPSiNPs as identified by LC-MS. Protein groups are organized according to protein functionality in the biological processes. The values of the adsorbed proteins are expressed as mean ± SD of the biological triplicates.

When nanoparticles come in contact with biological fluids, proteins rapidly adsorb to the nanoparticle surface conferring them a new biological identity [56]. In order to investigate protein adsorption on the model nanoparticles described above, the latter were incubated with either human blood serum or plasma for 1 h, (Figure 3A). The loosely bound proteins were removed by several washing steps (described in the methods section) and the nature and number of adsorbed proteins forming the so-called hard corona were analyzed. It is relevant to mention that both hard and soft protein corona exert an influence on the cellular response. However, while the soft protein corona presents a dynamic exchange, the hard protein corona rests strongly bound to the nanoparticles surface for longer time. Immune cells are therefore inevitably bound to interact with the hard corona over time [57].

The molecular weight and composition of the adsorbed protein corona was analyzed by sodium dodecyl sulfate-polyacrilamide gel electrophoresis (SDS-PAGE). The proteins isolated from the corona formed on the surface modified silica nanoparticles showed an intricate band pattern; this is evidence that the hard protein corona is a complex mix of proteins extending over a wide range of molecular weights and densities. Consistent with previous studies, the amount and nature of the proteins in the nanoparticles corona did not correlate with their relative abundance in the respective biological media, Figure 3B,C [58,59]. The major bands present in serum and plasma controls (e.g., red arrows) are not necessarily present with the same intensity on the nanoparticles’ corona.

The black arrows point to small molecular weights proteins present in the coronas that are not visible in the plasma and serum controls. Such discrepancy has been reported before and seems to be driven by electrostatic binding and steric effects linked to the nanoparticles physico-chemical properties [23]. We have previously studied the formation of serum protein corona on planar surfaces coated with comparable plasma polymers [52]. On these flat substrates, we reported a high concentration of large molecular weight proteins. In contrast, here in the corona forming around the model PPSiNPs, we see a clear enrichment in small molecular weight proteins. It can be argued that steric effect arising from the large curvature of the small nanoparticles favors the binding of smaller proteins compared to planar surfaces, thereby, leading to a different corona composition [60].

Quantification of the total amount of proteins adsorbed was performed by Pierce colorimetric assay as previously described (Figure S4) [61]. The data shows a correlation between the total amount of protein adsorbed and the sample negative charge. This correlation is in good agreement with previous reports [62].

For a detailed analysis, the hard protein corona extracted from the samples was digested with trypsin and the resulting peptides were analyzed by liquid chromatography mass spectrometry (LCMS). More than 283 and 286 proteins were identified in the hard corona of all five SiNPs and PPSiNPs in serum and plasma, respectively (Figure 3D,E and Figure S3). The complete list of detected proteins and grouping by category is provided in supporting information. Interestingly, AC functionalized particles presented the highest number of identified proteins in their corona (296 in serum and 294 in plasma). As depicted in the Venn diagrams, most of the proteins were common for all surface chemistries (Figure 3D,E). However, the AC and AA coated particles contained unique proteins not found on other surface chemistries and shared other proteins with only OX and OD.

Following established protocols [9], the proteins identified in the coronas were then grouped (Figure 3F,G) according to their functionality into 7 categories: (1) Albumin, which is the most abundant blood protein; (2) Acute phase proteins that play a role in the innate immune response; (3) Coagulation proteins, which are essential for blood clot formation; (4) Complement system proteins that contribute to opsonize pathogens and promote inflammation; (5) Immunoglobulins, which specifically recognize and bind antigens; (6) Lipoproteins involved in the transport of lipids; and (7) Tissue leakage proteins, which typically function within cells and other plasma components. The exact proteins assigned in these groups are listed in Table S1 provided in the supplementary information.

This classification highlighted patterns in the composition of the protein corona forming on the nanoparticles surface. The corona of unmodified SiNPs was enriched in coagulation factors: 60.28 µg/mg in serum and 76.07 µg/mg in plasma. However, it contained lower amounts of immunoglobulins (10.19 µg/mg in serum and 6.25 µg/mg in plasma) as well as albumin (8.51 µg/mg in serum and 5.59 µg/mg in plasma). AC particles had the highest amount of complement system proteins, binding 14.13 µg/mg in serum and 14.79 µg/mg in plasma. OD particles had the highest amount of albumin adsorbed 21.33 µg/mg in serum and 21.59 µg/mg in plasma. Interestingly, the percentage of lipoproteins bound in the hard corona increased with surface charge, adsorbing in larger amounts on the positively charged AA particles (41.56 µg/mg in serum and 42.89 µg/mg in plasma) and the least on negatively charged and hydrophilic AC particles (18.99 µg/mg in serum and 20.42 in plasma). In contrast, the percentage of coagulation factors proteins showed the opposite tendency, adsorbing in greater amount on hydrophilic, negatively charged AC particles (85.46 µg/mg in serum and 87.51 µg/mg in plasma) and lesser amount on AA (46.10 µg/mg in serum and 49.97 µg/mg in plasma). These trends were consistent across both plasma and serum media (Figure S5).

**Figure 4:**
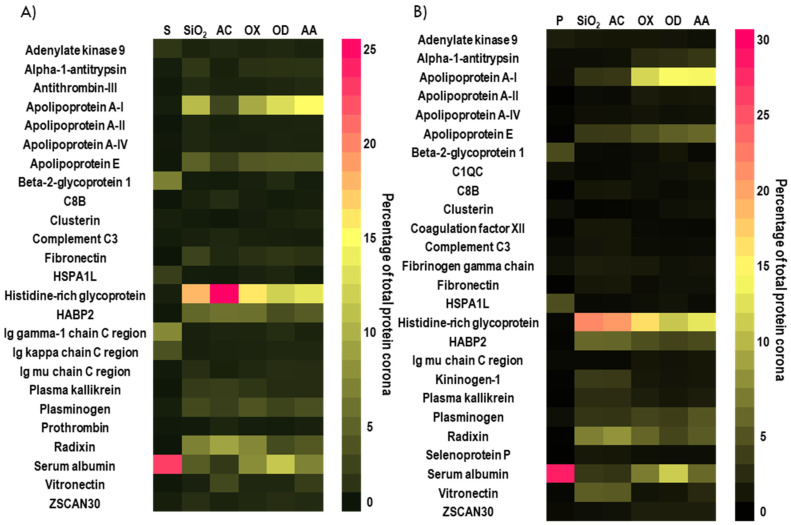
Heatmap of the most abundant protein in serum (**A**) and plasma (**B**) as well as the protein corona of the bare and PPSiNP determined by LC-MS. Values are calculated from the molar masses of each identified proteins. Only proteins that represent at least 1% of the protein mass on the nanoparticles’ corona are shown.

The proteins adsorbed on the bare and plasma coated nanoparticles, in abundance of at least 1%, are compiled in alphabetical order in the heatmaps presented in Figure 4A,B. As inferred from the SDS-PAGE data (Figure 3B,C), LCMS confirmed that the corona on bare and plasma coated nanoparticles contained small amounts of albumin (less than 12%) relative to the abundance of this protein in the compositions of serum and plasma (<22%). Furthermore, the protein corona of all samples was also enriched in Histidine-rich glycoprotein (HRG). However, the relative abundance of each protein was dependent on the properties of the plasma polymer coatings used for surface modification. Negatively charged AC coated particles had a higher affinity to HRG, which contributed 25% and 21% to the composition of serum and plasma coronas, respectively. The major components of OX coated particles corona were HRG (16% in serum,17% in plasma) and Apolipoprotein A-I (9% in serum, 12% in plasma). For OD coated particles, the most prominent proteins were Apolipoprotein A-I (13% in serum, 15% in plasma), HRG (12% in serum and plasma) and Albumin (11% in serum, 12% in plasma). Positively charged AA coated particles showed higher affinity to Apolipoprotein A-I (15% in serum and plasma) while HRG was determined at 13% in serum and 14% in plasma.

In order to understand how the nanoparticles physico-chemical properties govern the composition of the corona, the adsorbed proteins were sorted by increasing molecular weight (Figures S6 and S7) and increasing isoelectric points (Figures S8 and S9). We observed a distinct enrichment of proteins of small molecular weight (such as Apolipoprotein A-I, HGR and Albumin) on all nanoparticle coronas. In our previous study focused on protein adsorption on planar plasma polymer-coated substrates, one of the top five bound proteins was Apolipoprotein B-100 (MW = 517.0 KDa) [52]. In contrast, in the present study, the largest protein observed in the top five most abundant proteins adsorbed on PPSiNPs was Albumin, which is only 69.4 KDa in molecular weight. Hence, the present results indicate that protein adsorption on nanoparticles isn’t a process governed by chemistry alone, but that steric effects due to the particles curvature also influence the corona composition [63].

It is often suggested that positively charged proteins are attracted to negatively charged surfaces [64]. A study on model latex particles functionalized with different chemical groups has shown that negatively charged particles bearing acidic functional groups exhibit a general preference for proteins of isoelectric point greater than 5.5; while positively charged particles bearing amine functional groups principally bound proteins with isoelectric points smaller than 5.5 [65]. In agreement with this study, we found an increase of Apolipoprotein A-I (pI = 5.4) [66] adsorption to positively charged particles from both serum and plasma in the following order AA > OD > OX > AC. The opposite effect was noted for absorbed HRG protein (pI = 7.2) [66], which attached preferentially to silica particles with stronger negative charge and further followed the order AC > OX > OD > AA. Together these results show that both nanoparticles geometrical features and their unique chemical functionality direct protein adsorption and corona composition.

### 3.3. Effect of Plasma Polymer Surface Modification on Immunomodulatory Properties of Silica Nanoparticles

The immunogenicity of nanomaterials arises from their recognition by immune cells as foreign objects. The ensuing immune response includes the release of inflammatory cytokines, phagocytosis, reactive oxygen species overexpression, etc. [67]. The immune response to nanomaterials can be either enhanced or diminished by the protein corona that forms [68]. To determine the role of protein corona on the immunological response as a result of surface chemical modification, we examined the response of macrophages to bare SiNPs and PPSiNPs after protein corona formation. The first step was to ensure that the bare nanoparticles do not have cytotoxicity to human cells. We have previously reported the biocompatibility of the plasma polymer films deposited on flat substrates [52,69]. Here, we evaluated the cytotoxicity of SiNPs and PPSiNPs in culture of human fibroblasts which showed excellent biocompatibility (Figure S10). Macrophages are frequently chosen as model cells to study the immunomodulatory properties of nanomaterials in-vitro because these cells play a major role in our body’s immune response to the presence of a foreign material or pathogen. To test whether the protein corona adsorbed on PPSiNPs has an effect on cytokine production, human monocytes (THP-1) were differentiated into macrophages (dTHP-1) by addition of PMA. 

**Figure 5:**
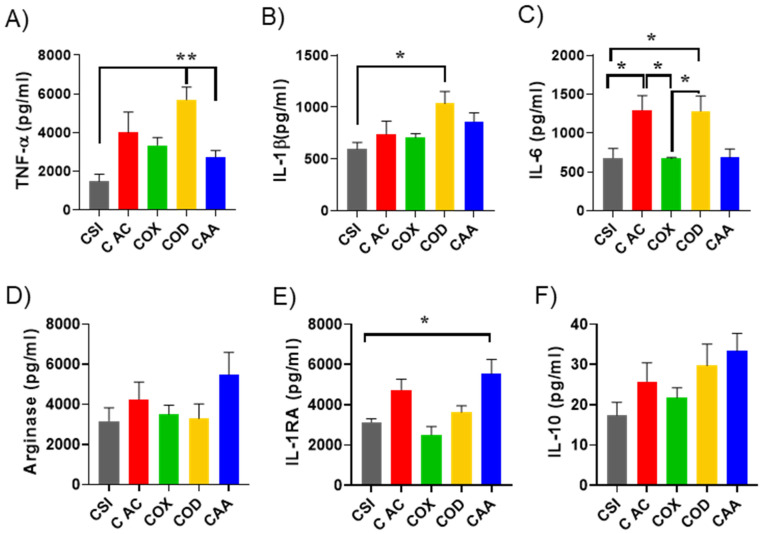
Inflammatory response of d-THP1 to PPSiNPs. Cytokine expression of pro-inflammatory cytokines (TNF-α, IL-6 and IL-1β) (**A**–**C**) and anti-inflammatory cytokines (Arginase, IL-1RA and IL-10) (**D**–**F**) in macrophage cultures exposed to nanoparticles-corona complexes from human plasma (CSi, CAC, COX, COD, CAA). Significance was tested by one-way ANOVA with Dunnett’s multiple comparisons. Error bars plotted are the standard error of the mean (n = 3). * *p* < 0.05, and ** *p* < 0.005.

Figure 5 shows the effect of the bare SiNPs and PPSiNPs on pro-inflammatory and anti-inflammatory cytokine production by lipopolysaccharides (LPS) activated macrophages. In the presence of protein corona, OD and AC plasma functionalized NP caused considerable changes in the production of pro-inflammatory cytokines compared to bare SiNPs. Significant differences were found between uncoated corona-SiNPs and corona-OD in terms of TNF-α (*p* < 0.005), IL6 (*p* < 0.05) and IL1b (*p* < 0.05) production, Figure 5A–C. OD-corona complexes also stimulated significantly greater secretion of TNFα pro-inflammatory cytokines compared to AA-corona complexes (*p* < 0.005), and more IL-6 than OX-corona complexes (*p* < 0.05). It is interesting to note that OD-corona nanoparticles, which had the lowest content of HRG protein in the corona (Figure 3F,G), also caused the strongest pro-inflammatory response. The enrichment of HRG protein on SiNP’s coronas has been shown to inhibit nanoparticles binding to the macrophage’s cellular membrane and their subsequent uptake [70]. The depletion of HGR adsorbed proteins on OD nanoparticles can cause an increased interaction of these particles with macrophages and stimulate the secretion of pro-inflammatory cytokines. In contrast, OD-corona nanoparticles had the highest albumin content. While albumin is known to have dysopsonin properties (stealth effect reducing inflammation), the strong pro-inflammatory response observed here indicates that the type of adsorbed protein is not the only influential factor. Structural changes adopted by the protein upon adsorption seems to also play an important role in the immune response. Indeed, structurally damaged albumin has been shown to be cleared from blood circulation by macrophages [71]. It is possible that albumin molecules unfolded as they bound to the OD coated nanoparticles, due to both the nanoparticles high hydrophobicity and their curvature. Previous studies have suggested that the presence of cryptic epitopes hidden within the albumin structure can be exposed during unfolding and be responsible for macrophage uptake [72].

Distinctively, AC-corona complexes also resulted in significant increase of IL-6 cytokine production (*p* < 0.05) compared to uncoated silica nanoparticles and OX-corona complexes. The protein corona of AC coated SiNPs was richer in complement proteins as shown in Figure 3F,G, (especially Complement C3 and Complement component C8 beta chain) compared to other PPSiNPs (Table S1).The presence of complement proteins in the corona has the capability to activate the complement system that is involved in the clearance of foreign entities [68]. Interestingly, Complement C3 has an active role in the three complement pathways (lectin, classical and alternative) that lead to the immune activation [73]. The high amount of adsorbed C3 protein on AC nanoparticles could explain the higher IL6 production, as C3 may lead to the activation of the complement system that ultimately causes the pro-inflammation responses detected here. On the other hand, AC also attracted less lipoproteins compared to the rest of the PPSiNPs. Lipoproteins have shown the stealth properties to reduce immune responses [61]. Together the increased amount of immune active complement proteins and the reduced affinity of stealth lipoproteins to the AC coating might have caused the observed elevated expression of in proinflammatory markers.

There were noticeable changes in the secretion of anti-inflammatory cytokines from d-THP1 cells upon exposure to nanoparticles having different surface modification (Figure 5D–F). These changes were not always significant but presented consistent trend. The increased expression of all three anti-inflammatory markers (Arginase, IL-1RA and IL-10) was most prominent in the case of AA functionalised PPSiNPs. An explanation of this finding could be linked to apolipoprotein A1, which is a major component of the corona formed on the AA modified PPSiNPs (15.1% serum and 14.6% plasma) and is well known to have anti-inflammatory properties [74]. In recent studies, Umemoto et al. [75] reported the anti-inflammatory effect of Apo A1 in macrophages and endothelial cells through cholesterol depletion of lipid rafts on the cellular membranes. Therefore, the high amount of apolipoprotein A1 in AA coronas could justify the increase on anti-inflammatory cytokine secretion after d-THP1 exposure to these particles.

An ideal nanocarrier must have dual properties, simultaneously enhancing the stability of the cargo and acting as immunological modulator. Herein, we used plasma polymerization that can help to modulate the nanocarrier properties. Our previous studies by Cavallaro et al. [30] showed the capability of plasma polymer coatings to be used in a single step solvent free encapsulation process for controlling drug release. This type of coatings can also be decorated by specific bioactive ligands to facilitate targeted delivery of therapeutics to desired location or for diagnostics purposes [34,76]. On another hand, coatings such as OD and AC can be potentially used to activate the immune response and to provide adjuvant properties to the nanocarriers in vaccine delivery. Overall, tailoring the surface chemistry on nanoscale objects in a single step is a challenge that plasma polymer deposition can overcome, making the process a promising utility to engineer the next generation of nanocarriers for delivery of vaccines and therapeutics.

## 4. Conclusions

The surface functionalization of nanomaterial is an important aspect of the development of effective nanocarrier for both drug and vaccine delivery, and diagnostics. In this work, a swift, one step, surfactant and solvent free approach was used to tailor the surface chemistry of model silica nanoparticles. We demonstrated that plasma polymerization successfully produced nano-thin films on the surface of nanomaterials, which presented distinctive chemical groups, namely carboxylic acid, oxazoline, amine, and hydrocarbons. This allowed us to evaluate the influence of nanomaterial surface chemistry on protein corona formation from blood serum and plasma. The different surface chemistry significantly influenced the composition of the protein coronas; however, they were all enriched in proteins with relatively small molecular weight, which we attributed to nanoparticles curvature. We also examined the immune responses invoked by the protein coronas formed on different surface chemistries. We found that nanoparticles containing surface chemistry dominated by hydrocarbon groups led to the formation of protein coronas rich in albumin and caused an increase in the production of pro-inflammatory cytokines from macrophages such as IL6 and TNFα. Nanoparticles presenting acid group on their surface also increase the production of these cytokines but in response to higher level of complement protein and lower amounts of lipoproteins found in their protein coronas. On another hand, amine rich coatings led to increased expressions of anti-inflammatory markers such as arginase. The findings demonstrate the potential to tailor the physiological responses to nanomaterials via engineering their surface chemical composition to suit a particular application. 

## Data Availability

The data presented in this study are available on request from the corresponding author.

## Supplementary Materials

The following supporting information can be downloaded at: https://www.mdpi.com/article/10.3390/nano12040682/s1, Figure S1: Principal component analysis of ToF-SIMS characterization data; Figure S2: Autofluorescence analysis of Plasma polymer coated silica nanoparticles, Figure S3: Relational table of the identified proteins in the plasma and serum coronas, Figure S4: Quantification of total proteins desorbed from the coronas, Figure S5: Relation of amount of coagulation proteins and lipoproteins adsorbed, Figure S6: Heat map of major protein components of serum protein corona formed on PPSiNPs sorted against increasing protein molecular weight (MW), Figure S7: Heat map of major protein components of plasma protein corona formed on PPSiNPs sorted against increasing protein molecular weight (MW), Figure S8: Heat map of major protein components of serum protein corona formed on PPSiNPs sorted against increasing isoelectric point (PI), Figure S9: Heat map of major protein components of plasma protein corona formed on PPSiNPs sorted against increasing isoelectric point (PI), Figure S10: Cell viability evaluation of SiNP, POx, OD, AC, AA samples at different concentrations for 24h on HFFs by resazurin assay. Table S1: List of all corona proteins identified by LC-MS. Average of the molar percentage of total protein unbound from each PPSiNPs type from plasma (P) and serum (S). References [77,78] are cited in the supplementary materials.
