# Supplementary material for: Nanoparticles Surface Chemistry Influence on Protein Corona Composition and Inflammatory Responses
Source: Nanomaterials (Basel). 2022 Feb 18;12(4):682. doi: 10.3390/nano12040682 (PMC8879273; doi:10.3390/nano12040682)
Supplement: Supplementary file 1 [file nanomaterials-12-00682-s001.zip › nanomaterials-1522521-supplementary.pdf]

Supplementary Materials

Nanoparticles Surface Chemistry Influence on Protein Corona Composition and Inflammatory Responses

Laura E. González-García [1], Melanie N. MacGregor [1,2,*], Rahul M. Visalakshan [1], Artur Lazarian [3], Alex A. Cavallaro [1], Svenja Morsbach [3], Agnieszka Mierczynska-Vasilev [4], Volker Mailänder [3,5], Katharina Landfester [3] and Krasimir Vasilev [1,6 *]

[1] UniSA STEM, Future Industries Institute, University of South Australia, Mawson Lakes, SA 5095, Australia; laura.gonzalez_garcia@mymail.unisa.edu.au (L.E.G.-G.); rahul.madathiparambil_visalakshan@mymail.unisa.edu.au (R.M.V.); alex-anthony.cavallaro@unisa.edu.au (A.A.C.)

[2] Flinders Institute for Nanoscale Science & Technology, College of Science and Engineering, Flinders University, Bedford Park, SA 5042, Australia

[3] Max Planck Institute for Polymer Research, Ackermannweg 10, 55128 Mainz, Germany; lazarian@mpip-mainz.mpg.de (A.L.); morsbachs@mpip-mainz.mpg.de (S.M.); mailaend@mpip-mainz.mpg.de (V.M.); landfest@mpip-mainz.mpg.de (K.L.)

[4] The Australian Wine Research Institute, Waite Precinct, Hartley Grove cnr Paratoo Road, Urrbrae, SA 5064, Australia; agnieszka.mierczynska@awri.com.au

[5] Department of Dermatology, University Medical Center of the Johannes Gutenberg-University Mainz, Langenbeckstr. 1, 55131 Mainz, Germany

[6] College of Medicine and Public Health, Flinders University, Sturt Road, Bedford Park, SA 5042, Australia

* Correspondence: melanie.macgregor@flinders.edu.au (M.N.M.); krasimir.vasilev@unisa.edu.au (K.V.)

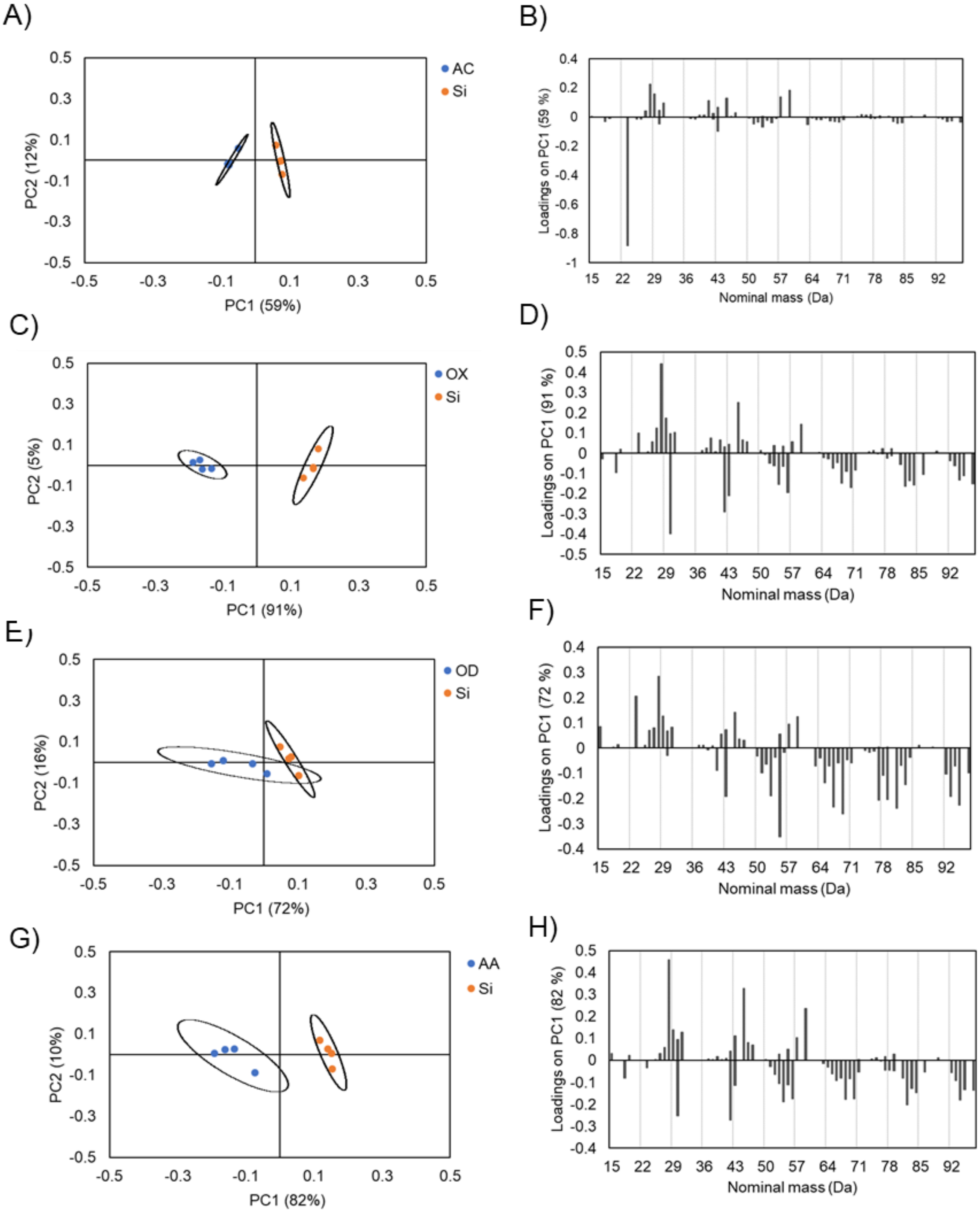

Figure S1. Principal component analysis of ToF-SIMS characterization data showing discrimination of bare SiO_2 and plasma polymer functionalities for AC A) OX C) OD E) and AA G). TOF-SIMS PC1 loadings for all fragments below 100m/z for AC B) OX D) OD F) and AA H).

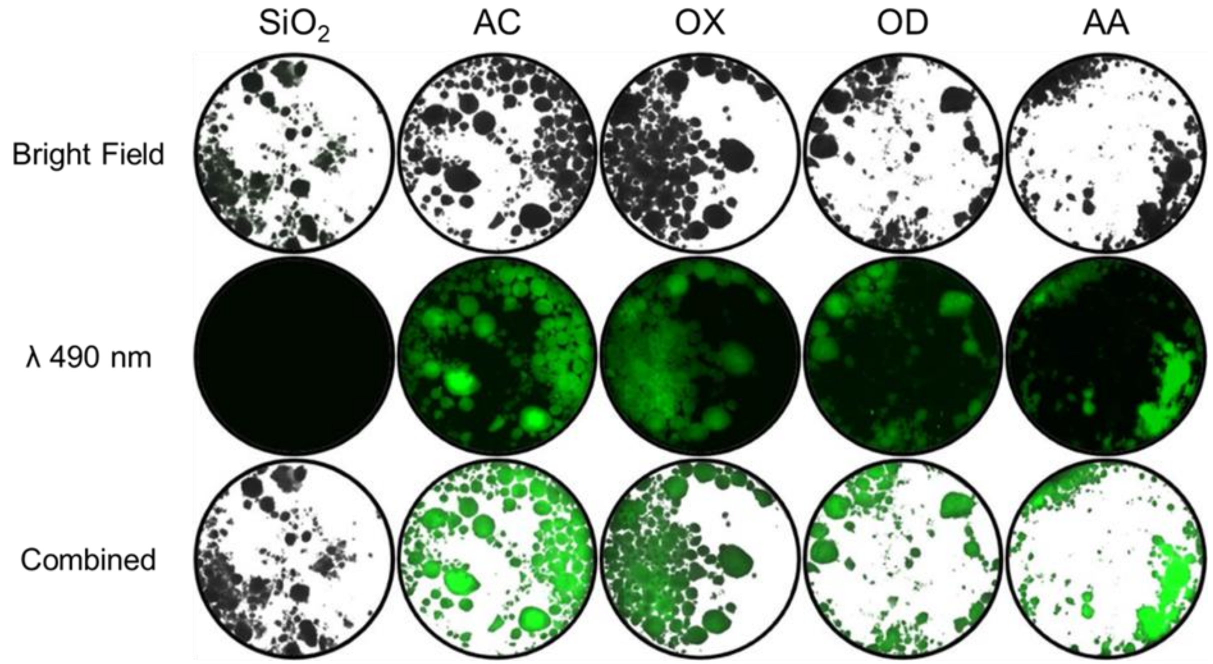

Figure S2. Autofluorescence analysis of Plasma polymer coated silica nanoparticles in bulk. Polymer coatings shown as green in signal channel and combined images.

The success of the nanoparticle functionalisation was first validated via fluorescence microscopy. Indeed, plasma polymers are rich in double bonds, a singular chemistry conferred by the ions and radicals present in the plasma phase during film deposition. The unsaturation and autofluorescence phenomena is related to the abstraction of radical H* from monomer precursors and subsequent radical self-quenching [77]. Plasma polymers autofluorescence has previously been used by others to determine the chemical stability of plasma coatings [78]. Fluorescence microscopy therefore constitute a swift approach to corroborate the presence of plasma polymer coatings on the nanoparticles. In bright field images, all nanoparticles appear black. Following excitation at 490 nm, the autofluorescence of bulk plasma modified particles SiO_2 nanoparticles (PPSiNP) is green, while the bare SiNP are not visible. The merged images of both optical channels shown in Figure S2 confirm the successful coating of the nanoparticles with AC, OX, OD and AA.

A)

	Si	AC	OX	OD	AA
Si					
AC	288 (97.3%)				
POX	287 (97.6%)	293 (99.0%)			
OD	288 (98.6%)	292 (98.6%)	290 (98.3%)		
AA	285 (97.9%)	287 (96.6%)	286 (96.9%)	287 (98.0%)	

B)

	Si	AC	OX	OD	AA
Si					
AC	288 (97.3%)				
POX	287 (97.6%)	293 (99.0%)			
OD	288 (98.6%)	292 (98.6%)	290 (98.3%)		
AA	285 (97.9%)	287 (96.6%)	286 (96.9%)	287 (98.0%)	

Figure S3. Relational table of the identified proteins in the plasma A) and serum B) coronas of all five bare and surface modified silica nanoparticles.

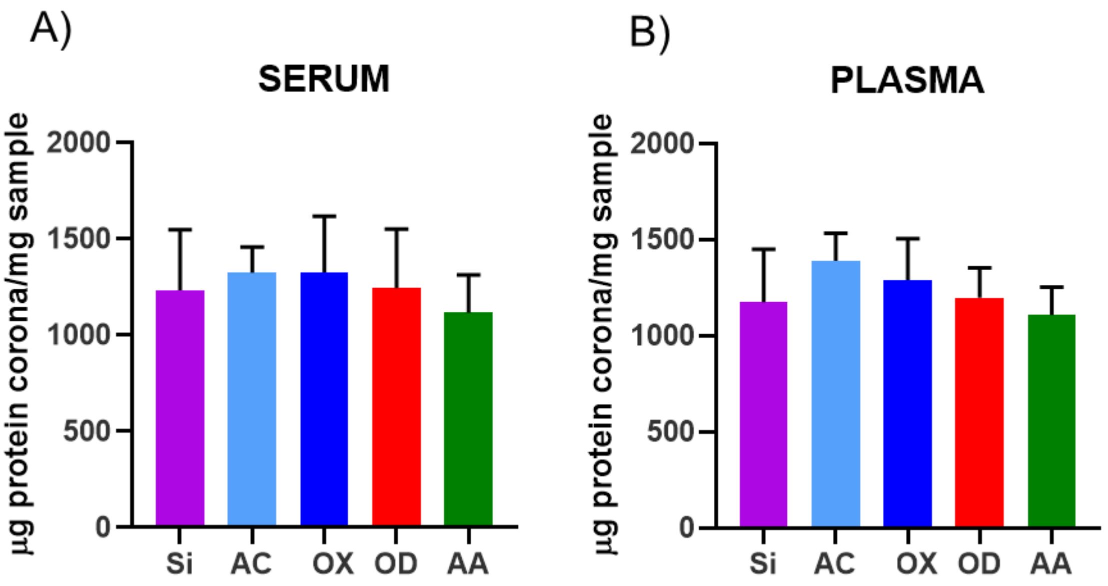

Figure S4. Total proteins desorbed from the coronas of the bare and modified silica nanoparticles quantified by Pierce assay on Serum A) and Plasma B).

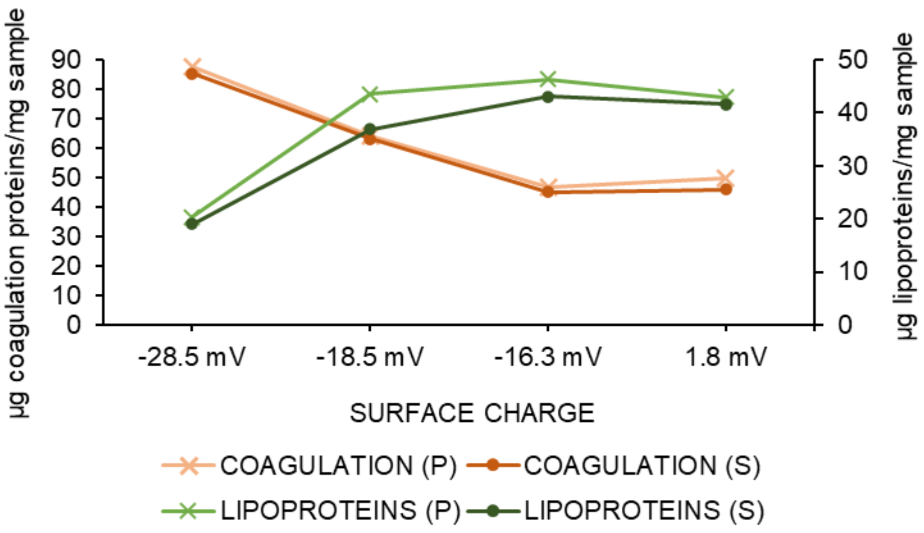

Figure S5. Relation of amount of coagulation proteins and lipoproteins adsorbed on the particles corona from plasma (P) and serum (S) with the nanoparticles surface charge.

PROTEIN	MW (KDa)	Serum	SiO_2	AC	OX	OD	AA
Apolipoprotein A-II	11.18	0.12	1.17	0.66	0.92	0.91	0.99
Ig kappa chain C region	11.77	3.89	0.69	0.93	0.94	1.17	1.21
Apolipoprotein A-I	30.78	0.54	10.61	3.14	9.54	12.83	15.14
Ig gamma-1 chain C region	36.11	7.44	0.75	1.02	1.07	1.48	0.93
Apolipoprotein E	36.15	0.25	4.89	2.71	4.29	4.49	4.52
Beta-2-glycoprotein 1	38.30	6.85	0.49	0.39	0.74	1.35	0.56
Apolipoprotein A-IV	45.40	0.32	1.18	0.83	0.76	0.93	0.87
Alpha-1-antitrypsin	46.74	0.52	2.18	0.77	1.77	1.86	1.98
Ig mu chain C region	49.44	0.35	1.63	0.79	1.26	1.59	1.53
Clusterin	52.50	0.72	0.41	0.25	0.58	0.86	1.11
Antithrombin-III	52.60	0.30	1.36	0.93	0.89	1.13	1.36
Vitronectin	54.31	0.23	0.82	3.29	1.21	1.17	2.62
ZSCAN30	56.36	0.63	1.66	0.88	1.26	1.60	1.38
Histidine-rich glycoprotein	59.58	0.73	17.88	25.31	15.94	12.08	13.37
Hyaluronan-binding protein 2	62.67	0.23	5.22	6.02	5.88	3.69	4.54
C8B	67.05	0.07	0.85	1.48	0.70	0.39	0.64
Radixin	68.56	0.09	6.98	9.20	7.41	3.46	4.34
Serum albumin	69.37	22.80	4.60	2.37	7.72	11.43	7.01
Prothrombin	70.04	0.27	0.35	1.09	0.52	0.48	0.75
Heat shock 70 kDa protein 1-like	70.38	2.63	0.46	0.43	0.82	1.12	0.40
Plasma kallikrein	71.37	0.16	2.50	2.64	2.14	1.49	1.52
Plasminogen	90.57	0.61	3.36	2.75	3.97	2.99	3.75
Complement C3	187.15	0.41	0.75	1.19	0.79	0.71	0.64
Adenylate kinase 9	221.41	2.16	0.97	1.30	1.05	1.23	0.90
Fibronectin	262.63	0.03	2.92	1.24	1.74	2.16	1.86

Colour scale: 25, 20, 15, 10, 5, 0

Figure S6. Heat map of major protein components of serum protein corona formed on PPSiNPs sorted against increasing protein molecular weight (MW).

PROTEIN	MW (KDa)	Plasma	SiO_2	AC	OX	OD	AA
Apolipoprotein A-II	11.18	0.04	0.62	0.51	1.48	1.17	1.27
C1QC	25.77	0.84	0.36	0.43	0.78	0.95	1.14
Apolipoprotein A-I	30.78	0.65	2.93	3.14	12.60	14.72	14.57
Apolipoprotein E	36.15	0.11	3.23	3.33	4.48	5.50	5.97
Beta-2-glycoprotein 1	38.30	4.30	0.52	0.46	0.70	1.16	0.47
Selenoprotein P	43.17	0.03	1.29	1.22	0.74	0.67	0.71
Apolipoprotein A-IV	45.40	0.25	0.83	0.82	1.12	0.88	0.99
Alpha-1-antitrypsin	46.74	0.67	0.70	0.87	2.36	2.58	3.11
Ig mu chain C region	49.44	0.44	0.43	0.46	1.15	1.01	1.20
Fibrinogen gamma chain	51.51	0.95	1.60	1.47	1.37	0.93	1.08
Clusterin	52.50	0.74	0.23	0.24	0.43	0.81	1.05
Vitronectin	54.31	0.30	5.37	5.12	1.05	1.16	2.20
ZSCAN30	56.36	0.54	0.76	0.92	1.65	1.60	1.57
Histidine-rich glycoprotein	59.58	0.32	22.05	21.01	16.96	11.72	13.58
Hyaluronan-binding protein 2	62.67	0.20	6.00	5.86	4.56	3.86	4.30
C8B	67.05	0.05	1.13	1.24	0.87	0.58	0.86
Coagulation factor XII	67.79	0.16	1.20	1.22	0.48	0.44	0.36
Radixin	68.56	0.07	7.38	8.58	5.88	3.95	5.21
Serum albumin	69.37	28.30	3.17	2.96	7.24	12.01	6.06
HSPA1L	70.38	4.49	0.47	0.49	0.71	1.07	0.54
Plasma kallikrein	71.37	0.10	2.44	2.46	1.62	1.20	1.57
Kininogen-1	71.96	0.17	3.30	3.27	1.27	1.07	1.22
Plasminogen	90.57	0.76	3.02	2.95	3.91	3.52	4.89
Complement C3	187.15	0.50	1.08	1.19	0.60	0.54	0.63
Adenylate kinase 9	221.41	1.59	1.27	1.18	1.15	1.10	0.77
Fibronectin	262.63	0.08	1.20	1.26	0.65	0.95	0.93

Color scale: 30, 25, 20, 15, 10, 5, 0

Figure S7. Heat map of major protein components of plasma protein corona formed on PPSiNPs sorted against increasing protein molecular weight (MW).

PROTEIN	PI	Serum	SiO_2	AC	OX	OD	AA
Adenylate kinase 9	4.8	2.16	0.97	1.30	1.05	1.23	0.90
Apolipoprotein A-II	4.9	0.12	1.17	0.66	0.92	0.91	0.99
Apolipoprotein A-IV	5.2	0.32	1.18	0.83	0.76	0.93	0.87
Alpha-1-antitrypsin	5.3	0.52	2.18	0.77	1.77	1.86	1.98
Fibronectin	5.4	0.03	2.92	1.24	1.74	2.16	1.86
Apolipoprotein A-I	5.4	0.54	10.61	3.14	9.54	12.83	15.14
Apolipoprotein E	5.5	0.25	4.89	2.71	4.29	4.49	4.52
Vitronectin	5.5	0.23	0.82	3.29	1.21	1.17	2.62
Ig kappa chain C region	5.6	3.89	0.69	0.93	0.94	1.17	1.21
Prothrombin	5.6	0.27	0.35	1.09	0.52	0.48	0.75
Heat shock 70 kDa protein 1-like	5.8	2.63	0.46	0.43	0.82	1.12	0.40
Serum albumin	5.9	22.80	4.60	2.37	7.72	11.43	7.01
Clusterin	6.0	0.72	0.41	0.25	0.58	0.86	1.11
Complement C3	6.1	0.41	0.75	1.19	0.79	0.71	0.64
Radixin	6.1	0.09	6.98	9.20	7.41	3.46	4.34
Hyaluronan-binding protein 2	6.2	0.23	5.22	6.02	5.88	3.69	4.54
Ig mu chain C region	6.3	0.35	1.63	0.79	1.26	1.59	1.53
Antithrombin-III	6.4	0.30	1.36	0.93	0.89	1.13	1.36
ZSCAN30	6.4	0.63	1.66	0.88	1.26	1.60	1.38
Plasminogen	6.9	0.61	3.36	2.75	3.97	2.99	3.75
Histidine-rich glycoprotein	7.2	0.73	17.88	25.31	15.94	12.08	13.37
Beta-2-glycoprotein 1	7.8	6.85	0.49	0.39	0.74	1.35	0.56
C8B	7.9	0.07	0.85	1.48	0.70	0.39	0.64
Plasma kallikrein	8.0	0.16	2.50	2.64	2.14	1.49	1.52
Ig gamma-1 chain C region	8.1	7.44	0.75	1.02	1.07	1.48	0.93

Color scale: 25, 20, 15, 10, 5, 0

Figure S8. Heat map of major protein components of serum protein corona formed on PPSiNPs sorted against increasing isoelectric point (PI). The protein analysis by PI shows no correlation to the protein corona.

PROTEIN	PI	Plasma	SiO_2	AC	OX	OD	AA
Adenylate kinase 9	4.8	1.59	1.27	1.18	1.15	1.10	0.77
Apolipoprotein A-II	4.9	0.04	0.62	0.51	1.48	1.17	1.27
Apolipoprotein A-IV	5.2	0.25	0.83	0.82	1.12	0.88	0.99
Fibrinogen gamma chain	5.2	0.95	1.60	1.47	1.37	0.93	1.08
Alpha-1-antitrypsin	5.3	0.67	0.70	0.87	2.36	2.58	3.11
Fibronectin	5.4	0.08	1.20	1.26	0.65	0.95	0.93
Apolipoprotein A-I	5.4	0.65	2.93	3.14	12.60	14.72	14.57
Apolipoprotein E	5.5	0.11	3.23	3.33	4.48	5.50	5.97
Vitronectin	5.5	0.30	5.37	5.12	1.05	1.16	2.20
HSPA1L	5.8	4.49	0.47	0.49	0.71	1.07	0.54
Serum albumin	5.9	28.30	3.17	2.96	7.24	12.01	6.06
Clusterin	6	0.74	0.23	0.24	0.43	0.81	1.05
Complement C3	6.1	0.50	1.08	1.19	0.60	0.54	0.63
Radixin	6.1	0.07	7.38	8.58	5.88	3.95	5.21
Hyaluronan-binding protein 2	6.2	0.20	6.00	5.86	4.56	3.86	4.30
Ig mu chain C region	6.3	0.44	0.43	0.46	1.15	1.01	1.20
ZSCAN30	6.4	0.54	0.76	0.92	1.65	1.60	1.57
Kininogen-1	6.5	0.17	3.30	3.27	1.27	1.07	1.22
Plasminogen	6.9	0.76	3.02	2.95	3.91	3.52	4.89
Histidine-rich glycoprotein	7.2	0.32	22.05	21.01	16.96	11.72	13.58
Coagulation factor XII	7.5	0.16	1.20	1.22	0.48	0.44	0.36
Selenoprotein P	7.7	0.03	1.29	1.22	0.74	0.67	0.71
Beta-2-glycoprotein 1	7.8	4.30	0.52	0.46	0.70	1.16	0.47
C8B	7.9	0.05	1.13	1.24	0.87	0.58	0.86
Plasma kallikrein	8	0.10	2.44	2.46	1.62	1.20	1.57
C1QC	8.3	0.84	0.36	0.43	0.78	0.95	1.14

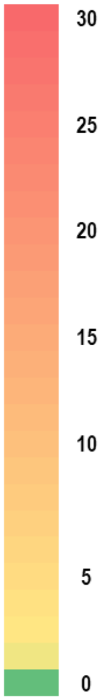

Figure S9. Heat map of major protein components of plasma protein corona formed on PPSiNPs sorted against increasing isoelectric point (PI). The protein analysis by PI shows no correlation to the protein corona.

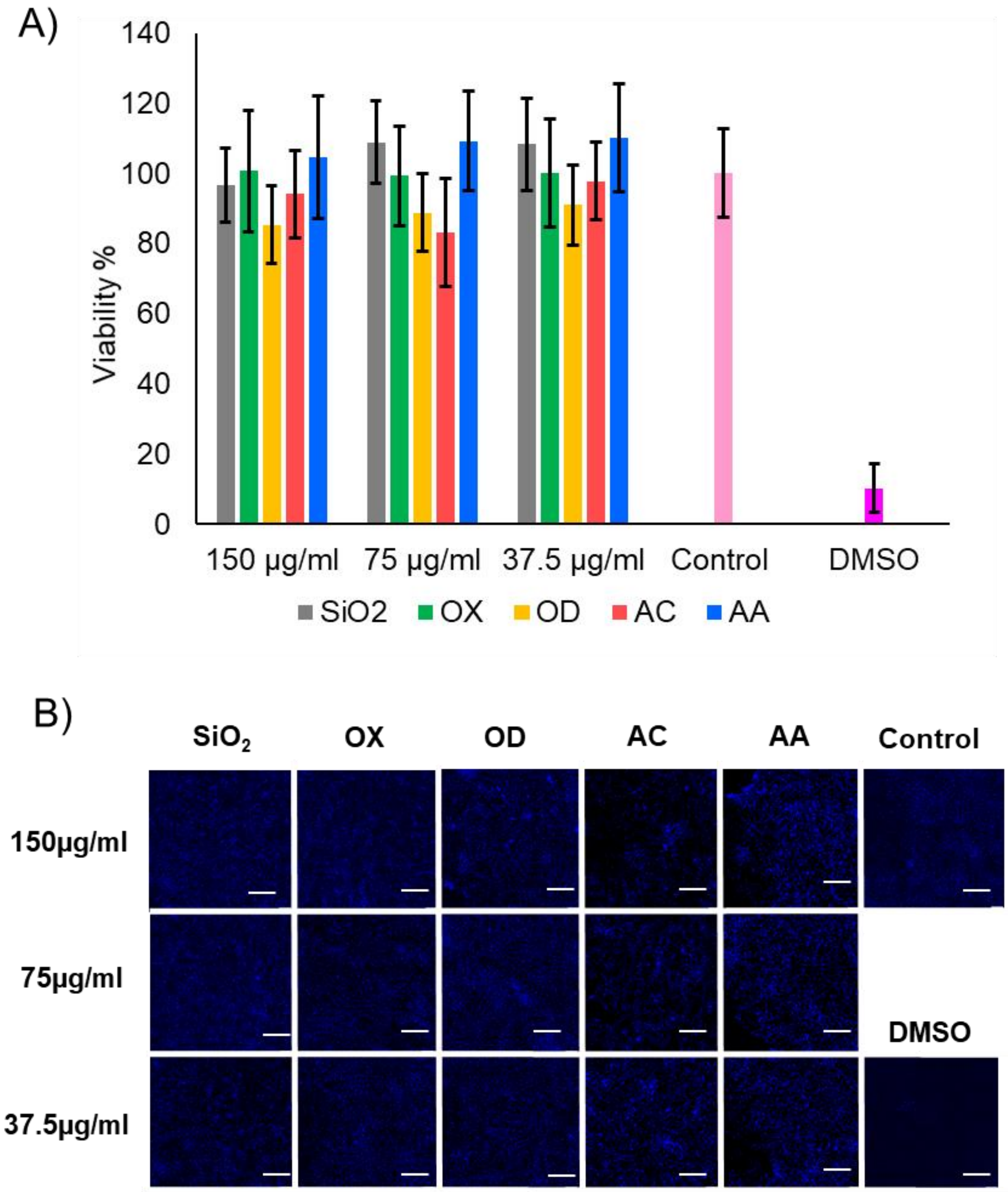

Figure S10. Cell viability evaluation of SiNP, POx, OD, AC, AA samples at different concentrations for 24 h on HFFs by resazurin assay A). Representative fluorescent microscopy images of cells after 24 h exposure to SiNP, POx, OD, AC, AA samples at different concentrations along with untreated and DMSO controls (scale bars 500 µm) B).

Table S1. List of all corona proteins identified by LC-MS. Average of the molar percentage of total protein unbound from each PPSiNPs type from plasma (P) and serum (S).

Annotation	Accession	Description	P	Si (P)	AC (P)	OX (P)	OD (P)	AA (P)	S	Si (S)	AC (S)	OX (S)	AA (S)	OD (S)
Acute Phase	P02751	Fibronectin	0.078	1.197	1.262	0.653	0.947	0.934	0.032	2.921	1.241	1.738	1.861	2.163
Acute Phase	P18428	Lipopolysaccharide-binding protein	0.021	0.209	0.200	0.036	0.088	0.040	0.045	0.068	0.259	0.087	0.086	0.204
Acute Phase	P01009	Alpha-1-antitrypsin	0.670	0.698	0.875	2.360	2.576	3.115	0.521	2.184	0.774	1.769	1.982	1.864
Acute Phase	P00738	Haptoglobin	1.408	0.089	0.081	0.105	0.194	0.102	0.765	0.153	0.064	0.179	0.136	0.303
Acute Phase	P02763	Alpha-1-acid glycoprotein 1	1.099	0.068	0.059	0.048	0.041	0.036	0.243	0.052	0.079	0.048	0.052	0.046
Acute Phase	P01011	Alpha-1-antichymotrypsin	0.260	0.017	0.016	0.016	0.018	0.016	0.205	0.013	0.023	0.016	0.021	0.022
Acute Phase	P01023	Alpha-2-macroglobulin	0.297	0.523	0.504	0.509	0.401	0.487	0.290	0.259	0.370	0.290	0.297	0.233
Acute Phase	P19652	Alpha-1-acid glycoprotein 2	0.204	0.095	0.087	0.098	0.116	0.097	0.184	0.117	0.098	0.157	0.111	0.132
Acute Phase	P02743	Serum amyloid P-component	0.110	0.034	0.033	0.029	0.028	0.030	0.127	0.048	0.177	0.036	0.033	0.043
Acute Phase	P02775	Platelet basic protein	0.050	0.049	0.049	0.023	0.026	0.021	0.291	0.022	0.037	0.027	0.025	0.031
Acute Phase	P02741	C-reactive protein	0.017	0.026	0.034	0.012	0.025	0.022	0.058	0.032	0.016	0.026	0.013	0.023
Acute Phase	P00450	Ceruloplasmin	0.174	0.018	0.021	0.028	0.025	0.032	0.110	0.021	0.020	0.020	0.026	0.028
Acute Phase	P35542	Serum amyloid A-4 protein	0.320	0.104	0.124	0.202	0.227	0.206	0.169	0.108	0.053	0.126	0.105	0.127
Acute Phase	P00739	Haptoglobin-related protein	0.229	0.111	0.104	0.111	0.132	0.130	0.302	0.179	0.097	0.174	0.198	0.184
Albumin	P02768	Serum albumin	28.305	3.168	2.958	7.244	12.013	6.058	22.803	4.600	2.367	7.724	7.013	11.433
Coagulation	P03952	Plasma kallikrein	0.099	2.444	2.457	1.620	1.199	1.566	0.160	2.498	2.636	2.142	1.521	1.490

Coagulation	Q14520	Hyaluronan-binding protein 2	0.198	6.004	5.864	4.557	3.864	4.295	0.229	5.215	6.016	5.882	4.538	3.686
Coagulation	P04196	Histidine-rich glycoprotein	0.318	22.055	21.008	16.960	11.719	13.575	0.732	17.884	25.305	15.943	13.369	12.079
Coagulation	P00747	Plasminogen	0.762	3.015	2.954	3.912	3.522	4.887	0.610	3.355	2.746	3.975	3.754	2.994
Coagulation	P00748	Coagulation factor XII	0.161	1.201	1.217	0.477	0.442	0.359	0.112	0.311	0.934	0.402	0.275	0.298
Coagulation	P05154	Plasma serine protease inhibitor	0.038	0.152	0.167	0.036	0.034	0.042	0.077	0.035	0.080	0.032	0.041	0.039
Coagulation	P01008	Antithrombin-III	0.283	0.218	0.232	0.876	0.872	0.853	0.300	1.356	0.926	0.889	1.359	1.133
Coagulation	P01042	Kininogen-1	0.169	3.301	3.272	1.272	1.071	1.216	0.427	0.213	0.687	0.297	0.252	0.220
Coagulation	P08697	Alpha-2-antiplasmin	0.082	0.168	0.151	0.083	0.099	0.086	0.215	0.143	0.165	0.164	0.177	0.150
Coagulation	P23142	Fibulin-1	0.047	0.079	0.083	0.072	0.063	0.064	0.062	0.089	0.072	0.088	0.078	0.064
Coagulation	Q13201	Multimerin-1	1.610	0.159	0.157	0.152	0.346	0.171	1.436	0.114	0.119	0.212	0.144	0.296
Coagulation	P35442	Thrombospondin-2	0.007	0.014	0.012	0.007	0.006	0.006	0.012	0.009	0.027	0.013	0.010	0.011
Coagulation	P03951	Coagulation factor XI	0.083	0.982	0.967	0.413	0.270	0.306	0.222	0.118	0.255	0.118	0.081	0.094
Coagulation	P02776	Platelet factor 4	0.073	0.033	0.033	0.030	0.036	0.031	0.108	0.097	0.598	0.088	0.127	0.140
Coagulation	P05546	Heparin cofactor 2	0.023	0.135	0.175	0.049	0.034	0.078	0.027	0.034	0.139	0.027	0.058	0.044
Coagulation	P02675	Fibrinogen beta chain	2.493	0.836	0.953	0.539	0.793	0.698	1.999	0.403	0.891	0.661	0.612	0.661
Coagulation	Q96IY4	Carboxypeptidase B2	0.051	0.056	0.051	0.037	0.030	0.033	0.070	0.083	0.107	0.088	0.117	0.104
Coagulation	P00734	Prothrombin	0.097	0.064	0.062	0.076	0.067	0.072	0.268	0.351	1.089	0.516	0.749	0.485
Coagulation	P02679	Fibrinogen gamma chain	0.948	1.604	1.471	1.369	0.929	1.080	0.108	0.039	0.029	0.033	0.063	0.041
Coagulation	P12259	Coagulation factor V	0.100	0.092	0.106	0.122	0.099	0.104	0.300	0.146	0.108	0.062	0.089	0.098
Coagulation	P02671	Fibrinogen alpha chain	0.717	0.501	0.542	0.568	0.409	0.527	0.252	0.095	0.079	0.096	0.086	0.101
Complement system	P07358	Complement component C8 beta chain	0.049	1.134	1.238	0.872	0.585	0.858	0.066	0.852	1.476	0.702	0.641	0.393
Complement system	P0C0L4	Complement C4-A	0.037	0.213	0.204	0.206	0.176	0.185	0.062	0.331	0.186	0.272	0.255	0.246
Complement system	P0C0L5	Complement C4-B	0.099	0.576	0.552	0.556	0.475	0.499	0.169	0.894	0.503	0.735	0.688	0.664

Complement system	P01024	Complement C3	0.498	1.079	1.192	0.596	0.537	0.633	0.406	0.746	1.195	0.792	0.642	0.711
Complement system	Q03591	Complement factor H-related protein 1	0.062	0.433	0.469	0.098	0.091	0.085	0.058	0.088	0.450	0.098	0.065	0.072
Complement system	P02748	Complement component C9	0.022	0.010	0.011	0.007	0.007	0.006	0.021	0.007	0.010	0.009	0.018	0.012
Complement system	P36980	Complement factor H-related protein 2	0.128	0.893	0.968	0.203	0.187	0.176	0.120	0.181	0.927	0.203	0.135	0.148
Complement system	P13671	Complement component C6	0.026	0.289	0.248	0.147	0.153	0.170	0.079	0.196	0.201	0.152	0.155	0.139
Complement system	P05155	Plasma protease C1 inhibitor	0.157	0.383	0.416	0.174	0.155	0.143	0.064	0.124	0.236	0.135	0.100	0.116
Complement system	P02730	Band 3 anion transport protein	0.000	0.000	0.000	0.000	0.000	0.000	0.000	0.000	0.000	0.000	0.000	0.000
Complement system	P02745	Complement C1q subcomponent subunit A	0.082	0.231	0.222	0.116	0.103	0.093	0.206	0.161	0.265	0.187	0.151	0.166
Complement system	P02747	Complement C1q subcomponent subunit C	0.839	0.358	0.429	0.782	0.946	1.137	0.617	0.806	0.520	0.713	0.874	0.987
Complement system	P07360	Complement component C8 gamma chain	0.013	0.028	0.024	0.014	0.011	0.011	0.011	0.012	0.030	0.019	0.016	0.016
Complement system	P00751	Complement factor B	0.017	0.149	0.148	0.055	0.057	0.075	0.075	0.048	0.047	0.030	0.044	0.040
Complement system	P04003	C4b-binding protein alpha chain	0.132	0.077	0.084	0.108	0.092	0.120	0.063	0.196	0.084	0.181	0.182	0.153
Complement system	P09871	Complement C1s subcomponent	0.088	0.077	0.081	0.156	0.190	0.173	0.381	0.175	0.102	0.139	0.172	0.171
Complement system	P00736	Complement C1r subcomponent	0.034	0.007	0.006	0.008	0.006	0.007	0.018	0.006	0.008	0.004	0.006	0.005

Complement system	Q9BXR6	Complement factor H-related protein 5	1.386	0.181	0.198	0.267	0.395	0.253	0.802	0.249	0.180	0.301	0.222	0.394
Complement system	P02746	Complement C1q subcomponent subunit B	0.266	0.237	0.236	0.084	0.091	0.050	0.360	0.112	0.323	0.162	0.112	0.159
Complement system	P00746	Complement factor D	0.026	0.024	0.023	0.023	0.021	0.021	0.043	0.027	0.022	0.025	0.032	0.029
Complement system	P08603	Complement factor H	0.920	0.314	0.338	0.468	0.473	0.406	0.420	0.322	0.344	0.333	0.233	0.351
Immunoglobulins	P01764 P01768	Immunoglobulin heavy variable 3-23	0.020	0.003	0.003	0.004	0.004	0.003	0.044	0.004	0.003	0.004	0.006	0.006
Immunoglobulins	P0CG05 A0M8Q6 P0CF74 P0CG04 P0CG06	Ig lambda-2 chain C regions	0.294	0.060	0.051	0.039	0.039	0.030	0.994	0.052	0.076	0.093	0.100	0.104
Immunoglobulins	P01876	Ig alpha-1 chain C region	1.809	0.493	0.483	0.557	0.473	0.499	2.989	0.555	0.674	0.661	0.772	0.690
Immunoglobulins	P01871	Ig mu chain C region	0.445	0.427	0.463	1.153	1.009	1.199	0.354	1.630	0.786	1.262	1.528	1.593
Immunoglobulins	A0A0C4DH72 A0A0C4DH73 P01597 P01599 P01611 P04432	Immunoglobulin kappa variable 1-6	0.116	0.011	0.012	0.011	0.022	0.011	0.108	0.026	0.029	0.025	0.031	0.034
Immunoglobulins	P01624 A0A0C4DH55	Immunoglobulin kappa variable 3-15	0.075	0.026	0.028	0.021	0.022	0.020	0.137	0.031	0.023	0.030	0.032	0.031
Immunoglobulins	P01825 A0A0C4DH41 P01824 P06331	Immunoglobulin heavy variable 4-59	0.086	0.112	0.144	0.132	0.169	0.188	0.099	0.199	0.098	0.137	0.132	0.140

Immunoglobulins	P01767	Immunoglobulin heavy variable 3-53	0.037	0.022	0.018	0.012	0.012	0.011	0.145	0.014	0.035	0.014	0.020	0.022
Immunoglobulins	P01877	Ig alpha-2 chain C region	0.167	0.109	0.091	0.094	0.070	0.076	0.234	0.098	0.110	0.077	0.105	0.095
Immunoglobulins	P01594 P01593	Immunoglobulin kappa variable 1-33	0.051	0.000	0.000	0.001	0.000	0.000	0.004	0.000	0.003	0.000	0.002	0.002
Immunoglobulins	P04220	Ig mu heavy chain disease protein	0.174	0.243	0.237	0.255	0.313	0.295	0.408	0.556	0.418	0.624	0.885	0.980
Immunoglobulins	A0A075B6P5 P01615	Immunoglobulin kappa variable 2-28	0.411	0.068	0.069	0.046	0.060	0.055	0.534	0.090	0.069	0.096	0.157	0.139
Immunoglobulins	P01717	Immunoglobulin lambda variable 3-25	0.040	0.033	0.033	0.024	0.026	0.028	0.170	0.016	0.033	0.030	0.023	0.031
Immunoglobulins	A0A0B4J1X5 A0A075B6Q5 A0A0C4DH42 P01772	Immunoglobulin heavy variable 3-74	0.002	0.000	0.000	0.001	0.001	0.001	0.007	0.001	0.001	0.001	0.001	0.001
Immunoglobulins	P04433 A0A0A0MRZ8	Immunoglobulin kappa variable 3-11	0.112	0.161	0.138	0.044	0.049	0.087	0.115	0.056	0.117	0.069	0.138	0.074
Immunoglobulins	P01718	Immunoglobulin lambda variable 3-27	0.018	0.009	0.008	0.019	0.019	0.021	0.027	0.039	0.019	0.034	0.034	0.042
Immunoglobulins	P01880	Ig delta chain C region	0.002	0.036	0.026	0.031	0.015	0.019	0.046	0.028	0.052	0.031	0.027	0.020
Immunoglobulins	A0A0A0MS15	Immunoglobulin heavy variable 3-49	0.001	0.000	0.000	0.000	0.000	0.000	0.004	0.000	0.000	0.000	0.001	0.000
Immunoglobulins	A0A0C4DH38	Immunoglobulin heavy variable 5-51	0.046	0.028	0.029	0.021	0.023	0.020	0.051	0.017	0.033	0.024	0.026	0.029
Immunoglobulins	P01591	Immunoglobulin J chain	0.028	0.018	0.019	0.013	0.016	0.016	0.046	0.019	0.018	0.024	0.028	0.028
Immunoglobulins	A0A0B4J1V1	Immunoglobulin heavy variable 3-21	0.059	0.006	0.006	0.007	0.008	0.007	0.036	0.013	0.014	0.016	0.020	0.018
Immunoglobulins	P01857	Ig gamma-1 chain C region	5.482	0.743	0.768	0.584	0.594	0.476	7.441	0.750	1.020	1.067	0.929	1.477

Immunoglobulins	P01619 A0A0C4DH25	Immunoglobulin kappa variable 3-20	0.140	0.043	0.042	0.034	0.034	0.035	0.107	0.061	0.058	0.066	0.071	0.072
Immunoglobulins	P01860	Ig gamma-3 chain C region	0.491	0.188	0.185	0.123	0.117	0.089	0.301	0.137	0.247	0.211	0.187	0.256
Immunoglobulins	P01861	Ig gamma-4 chain C region	0.057	0.023	0.023	0.023	0.019	0.023	0.016	0.012	0.015	0.017	0.022	0.020
Immunoglobulins	P01859	Ig gamma-2 chain C region	0.905	0.130	0.125	0.080	0.081	0.065	0.352	0.239	0.299	0.314	0.438	0.362
Immunoglobulins	P01834	Ig kappa chain C region	3.142	0.483	0.458	0.426	0.420	0.316	3.892	0.694	0.929	0.936	1.207	1.171
Immunoglobulins	P01780 A0A0B4J1X8 P01762 P01763 P01782	Immunoglobulin heavy variable 3-7	0.096	0.018	0.021	0.020	0.020	0.021	0.077	0.043	0.038	0.048	0.062	0.058
Immunoglobulins	A0A075B6I0	Immunoglobulin lambda variable 8-61	1.188	0.051	0.050	0.048	0.190	0.079	1.038	0.128	0.025	0.147	0.101	0.324
Lipoproteins	P02649	Apolipoprotein E	0.107	3.231	3.326	4.476	5.500	5.969	0.247	4.891	2.713	4.285	4.519	4.485
Lipoproteins	P02647 Q9P2D0	Apolipoprotein A	0.654	2.928	3.143	12.596	14.719	14.569	0.543	10.608	3.140	9.541	15.139	12.826
Lipoproteins	O14791	Apolipoprotein L1	0.045	0.088	0.088	0.159	0.162	0.181	0.045	0.149	0.089	0.144	0.191	0.167
Lipoproteins	P02652	Apolipoprotein A-II	0.044	0.625	0.512	1.475	1.168	1.272	0.117	1.175	0.659	0.919	0.986	0.914
Lipoproteins	Q6Q788	Apolipoprotein A-V	0.058	0.092	0.098	0.137	0.074	0.094	0.032	0.205	0.246	0.138	0.078	0.083
Lipoproteins	P02749	Beta-2-glycoprotein 1	4.301	0.517	0.464	0.697	1.160	0.472	6.853	0.494	0.388	0.745	0.556	1.351
Lipoproteins	P10909	Clusterin	0.737	0.227	0.242	0.434	0.814	1.046	0.718	0.410	0.245	0.576	1.115	0.856
Lipoproteins	P06727	Apolipoprotein A-IV	0.246	0.832	0.817	1.122	0.881	0.993	0.320	1.175	0.828	0.763	0.872	0.934
Lipoproteins	P27169	Serum paraoxonase/arylesterase 1	0.271	0.121	0.108	0.248	0.298	0.265	0.086	0.259	0.137	0.329	0.363	0.354
Lipoproteins	P05090	Apolipoprotein D	0.015	0.099	0.100	0.103	0.077	0.083	0.085	0.126	0.147	0.120	0.109	0.102

Lipoproteins	O95445	Apolipoprotein M	0.026	0.025	0.027	0.056	0.058	0.058	0.040	0.055	0.036	0.062	0.066	0.054
Lipoproteins	P08519	Apolipoprotein(a)	0.023	0.048	0.046	0.041	0.039	0.046	0.072	0.032	0.030	0.051	0.058	0.069
Lipoproteins	P04114	Apolipoprotein B-100	1.511	0.741	0.774	0.907	0.807	0.708	0.856	0.915	0.897	0.889	0.734	0.858
Lipoproteins	P55056	Apolipoprotein C-IV	0.016	0.043	0.039	0.052	0.037	0.033	0.149	0.004	0.007	0.005	0.008	0.011
Other Plasma components	Q9NV56	MRG/MORF4L-binding protein	0.004	0.028	0.026	0.020	0.016	0.017	0.007	0.030	0.028	0.022	0.016	0.016
Other Plasma components	P49908	Selenoprotein P	0.033	1.293	1.218	0.735	0.671	0.711	0.078	0.899	0.834	0.906	0.727	0.684
Other Plasma components	P35241	Radixin	0.067	7.382	8.577	5.881	3.954	5.211	0.095	6.980	9.200	7.410	4.339	3.464
Other Plasma components	Q8IWL2	Pulmonary surfactant-associated protein A1	0.032	0.003	0.005	0.000	0.004	0.001	0.060	0.001	0.002	0.004	0.001	0.005
Other Plasma components	P25311	Zinc-alpha-2-glycoprotein	0.074	0.007	0.006	0.003	0.002	0.001	0.097	0.002	0.007	0.002	0.002	0.003
Other Plasma components	Q8IUC0	Keratin-associated protein 13-1	0.000	0.009	0.002	0.051	0.040	0.059	0.001	0.009	0.011	0.027	0.086	0.037
Other Plasma components	P82970	High mobility group nucleosome-binding domain-containing protein 5	0.000	0.138	0.165	0.113	0.044	0.097	0.001	0.069	0.272	0.097	0.046	0.040
Other Plasma components	P22352	Glutathione peroxidase 3	0.008	0.008	0.009	0.020	0.019	0.034	0.010	0.013	0.013	0.026	0.046	0.020
Other Plasma components	Q86W11	Zinc finger and SCAN domain-containing protein 30	0.535	0.758	0.923	1.646	1.595	1.570	0.631	1.658	0.884	1.260	1.378	1.599
Other Plasma components	P36955	Pigment epithelium-derived factor	0.017	0.096	0.091	0.024	0.024	0.020	0.029	0.028	0.057	0.026	0.020	0.029
Other Plasma components	Q99497	Protein deglycase DJ-1	0.053	0.008	0.006	0.015	0.027	0.009	0.058	0.007	0.003	0.014	0.008	0.026

Other Plasma components	P57796	Calcium-binding protein 4	0.005	0.083	0.073	0.209	0.107	0.106	0.030	0.112	0.114	0.082	0.099	0.069
Other Plasma components	Q96E39	RNA binding motif protein, X-linked-like-1	0.006	0.018	0.022	0.027	0.034	0.050	0.015	0.050	0.025	0.060	0.050	0.044
Other Plasma components	A6NFZ4	Protein FAM24A	0.141	0.302	0.201	0.234	0.208	0.296	0.064	0.447	0.151	0.510	0.252	0.318
Other Plasma components	Q99969	Retinoic acid receptor responder protein 2	0.036	0.199	0.191	0.092	0.089	0.075	0.070	0.058	0.124	0.049	0.057	0.060
Other Plasma components	P13639	Elongation factor 2	0.004	0.032	0.043	0.115	0.144	0.168	0.006	0.069	0.082	0.109	0.116	0.083
Other Plasma components	Q96QA5	Gasdermin-A	0.072	0.472	0.494	0.634	0.535	0.793	0.403	0.450	0.510	0.617	0.618	0.347
Other Plasma components	Q6ZS10	C-type lectin domain family 17, member A	0.386	0.118	0.135	0.309	0.411	0.428	0.458	0.208	0.109	0.196	0.322	0.314
Other Plasma components	P11142 P54652	Heat shock cognate 71 kDa protein	0.021	0.081	0.089	0.161	0.140	0.152	0.059	0.181	0.126	0.105	0.119	0.112
Other Plasma components	Q01469 A8MUU1	Fatty acid-binding protein, epidermal	0.037	0.011	0.012	0.012	0.018	0.014	0.084	0.011	0.015	0.012	0.010	0.018
Other Plasma components	P09211	Glutathione S-transferase P	0.173	0.005	0.005	0.016	0.013	0.011	0.122	0.028	0.016	0.019	0.013	0.030
Other Plasma components	P31040	Succinate dehydrogenase [ubiquinone] flavoprotein subunit, mitochondrial	0.000	0.057	0.044	0.000	0.073	0.041	0.014	0.099	0.001	0.127	0.074	0.090
Other Plasma components	P23284	Peptidyl-prolyl cis-trans isomerase B	0.010	0.009	0.010	0.005	0.008	0.007	0.053	0.003	0.007	0.002	0.005	0.007
Other Plasma components	P02790	Hemopexin	0.517	0.187	0.188	0.097	0.115	0.111	0.632	0.096	0.181	0.107	0.109	0.118

Other Plasma components	P19827	Inter-alpha-trypsin inhibitor heavy chain H1	0.148	0.044	0.043	0.033	0.023	0.023	0.109	0.032	0.025	0.040	0.029	0.056
Other Plasma components	Q5T700	Low-density lipoprotein receptor class A domain-containing protein 1	0.050	0.506	0.662	0.614	0.322	0.416	0.033	0.489	0.747	0.163	0.345	0.332
Other Plasma components	P04217	Alpha-1B-glycoprotein	0.097	0.253	0.252	0.654	0.736	0.940	0.119	0.936	0.305	0.706	0.898	0.746
Other Plasma components	P24844	Myosin regulatory light polypeptide 9	0.010	0.046	0.038	0.027	0.028	0.013	0.012	0.019	0.042	0.044	0.050	0.042
Other Plasma components	Q9BQE6	LBH domain-containing protein 1	0.000	0.004	0.006	0.007	0.007	0.009	0.009	0.002	0.008	0.007	0.009	0.005
Other Plasma components	P24592	Insulin-like growth factor-binding protein 6	0.033	0.060	0.082	0.183	0.187	0.177	0.056	0.184	0.081	0.133	0.156	0.169
Other Plasma components	P53634	Dipeptidyl peptidase 1	0.103	0.002	0.001	0.002	0.004	0.001	0.181	0.010	0.003	0.004	0.011	0.021
Other Plasma components	Q6ZVL6	UPF0606 protein KIAA1549L	0.111	0.126	0.135	0.086	0.100	0.084	0.137	0.160	0.200	0.109	0.107	0.124
Other Plasma components	P22528 P35321	Cornifin-B	0.051	0.131	0.131	0.067	0.048	0.086	0.031	0.052	0.128	0.040	0.063	0.035
Other Plasma components	P02750	Leucine-rich alpha-2-glycoprotein	0.010	0.000	0.000	0.000	0.000	0.000	0.019	0.000	0.000	0.000	0.000	0.000
Other Plasma components	Q08345	Epithelial discoidin domain-containing receptor 1	0.029	0.003	0.004	0.002	0.006	0.002	0.025	0.004	0.001	0.005	0.002	0.006
Other Plasma components	P07339	Cathepsin D	0.019	0.143	0.160	0.126	0.089	0.105	0.135	0.122	0.203	0.156	0.146	0.090
Other Plasma components	Q99687	Homeobox protein Meis3	0.009	0.046	0.039	0.009	0.009	0.009	0.017	0.015	0.047	0.013	0.012	0.013

Other Plasma components	Q8N8B7	Transcription elongation factor A N-terminal and central domain-containing protein	0.148	0.003	0.002	0.000	0.001	0.001	0.061	0.000	0.001	0.002	0.000	0.000
Other Plasma components	P48594	Serpin B4	0.460	0.089	0.086	0.195	0.246	0.203	0.644	0.245	0.095	0.215	0.193	0.231
Other Plasma components	P07355 A6NMY6	Annexin A2	0.003	0.002	0.002	0.003	0.002	0.003	0.013	0.001	0.002	0.001	0.001	0.001
Other Plasma components	P39060	Collagen alpha-1(XVIII) chain	0.008	0.093	0.085	0.071	0.054	0.067	0.093	0.098	0.043	0.055	0.061	0.038
Other Plasma components	P10599	Thioredoxin	0.000	0.007	0.010	0.012	0.035	0.039	0.008	0.079	0.002	0.091	0.097	0.096
Other Plasma components	P84077 P18085 P61204 P84085	ADP-ribosylation factor 1	0.005	0.003	0.003	0.002	0.003	0.002	0.025	0.007	0.002	0.002	0.005	0.008
Other Plasma components	Q86UC2	Radial spoke head protein 3 homolog	0.000	0.002	0.000	0.000	0.000	0.000	0.000	0.000	0.005	0.001	0.000	0.000
Other Plasma components	P02774	Vitamin D-binding protein	1.284	0.192	0.193	0.383	0.410	0.348	1.605	0.306	0.260	0.394	0.434	0.464
Other Plasma components	O15182	Centrin-3	0.001	0.013	0.023	0.000	0.008	0.003	0.007	0.004	0.005	0.007	0.002	0.011
Other Plasma components	P10646	Tissue factor pathway inhibitor	0.003	0.011	0.010	0.005	0.003	0.004	0.012	0.001	0.008	0.001	0.001	0.001
Other Plasma components	O14960	Leukocyte cell-derived chemotaxin-2	0.047	0.259	0.286	0.093	0.105	0.141	0.142	0.080	0.132	0.097	0.079	0.068
Other Plasma components	O75381	Peroxisomal membrane protein PEX14	0.000	0.014	0.011	0.000	0.001	0.004	0.000	0.001	0.020	0.003	0.014	0.000

Other Plasma components	O15335	Chondroadherin	0.001	0.010	0.006	0.003	0.002	0.001	0.014	0.005	0.007	0.001	0.004	0.001
Other Plasma components	P01019	Angiotensinogen	0.179	0.056	0.045	0.046	0.056	0.033	0.169	0.073	0.068	0.079	0.066	0.084
Other Plasma components	Q8NEM0	Microcephalin	0.015	0.040	0.043	0.016	0.013	0.012	0.031	0.016	0.028	0.021	0.008	0.009
Other Plasma components	P61626	Lysozyme C	0.002	0.053	0.049	0.014	0.009	0.005	0.020	0.007	0.025	0.003	0.002	0.004
Other Plasma components	P68104 Q05639 Q5VTE0	Elongation factor 1-alpha 1	0.004	0.004	0.005	0.003	0.004	0.004	0.018	0.003	0.003	0.002	0.004	0.004
Other Plasma components	P62979 P0CG47 P0CG48 P62987	Ubiquitin-40S ribosomal protein S27a	0.024	0.080	0.084	0.082	0.074	0.097	0.048	0.054	0.073	0.059	0.076	0.057
Other Plasma components	P49711	Transcriptional repressor CTCF	0.037	0.003	0.006	0.009	0.007	0.007	0.063	0.003	0.016	0.002	0.002	0.006
Other Plasma components	P43652	Afamin	0.014	0.090	0.106	0.104	0.149	0.157	0.180	0.141	0.107	0.099	0.137	0.121
Other Plasma components	Q9UKZ9	Procollagen C-endopeptidase enhancer 2	0.013	0.002	0.002	0.001	0.001	0.001	0.015	0.003	0.002	0.002	0.001	0.002
Other Plasma components	P27482	Calmodulin-like protein 3	0.032	0.053	0.048	0.017	0.016	0.027	0.021	0.014	0.045	0.015	0.037	0.016
Other Plasma components	Q02325	Plasminogen-like protein B	0.000	0.002	0.003	0.001	0.004	0.004	0.006	0.014	0.000	0.003	0.003	0.010
Other Plasma components	P02787	Serotransferrin	1.454	0.419	0.410	0.350	0.378	0.348	1.646	0.180	0.183	0.197	0.166	0.258

Other Plasma components	Q5VSP4	Putative lipocalin 1-like protein 1	0.031	0.004	0.004	0.015	0.020	0.013	0.044	0.007	0.003	0.010	0.011	0.021
Other Plasma components	Q13705	Activin receptor type-2B	0.000	0.014	0.014	0.006	0.007	0.007	0.013	0.003	0.011	0.004	0.011	0.008
Other Plasma components	Q9NX94	WW domain binding protein 1-like	0.154	0.038	0.032	0.146	0.062	0.047	0.078	0.016	0.020	0.025	0.021	0.039
Other Plasma components	P17936	Insulin-like growth factor-binding protein 3	0.023	0.054	0.059	0.037	0.032	0.046	0.017	0.023	0.047	0.037	0.042	0.031
Other Plasma components	P28300	Protein-lysine 6-oxidase	0.008	0.001	0.000	0.000	0.004	0.000	0.007	0.001	0.001	0.000	0.000	0.000
Other Plasma components	P04080	Cystatin-B	0.007	0.003	0.003	0.002	0.003	0.003	0.008	0.002	0.003	0.002	0.003	0.004
Other Plasma components	P68871 P02100 P69891 P69892	Hemoglobin subunit beta	0.043	0.025	0.025	0.020	0.020	0.025	0.038	0.009	0.008	0.020	0.046	0.036
Other Plasma components	P14923	Junction plakoglobin	0.001	0.010	0.002	0.000	0.000	0.000	0.004	0.000	0.014	0.002	0.002	0.001
Other Plasma components	Q7Z5L7	Podocan	0.000	0.001	0.001	0.000	0.000	0.000	0.000	0.000	0.000	0.000	0.000	0.000
Other Plasma components	Q8IUD6	E3 ubiquitin-protein ligase RNF135	0.000	0.002	0.001	0.001	0.000	0.000	0.002	0.001	0.001	0.001	0.001	0.000
Other Plasma components	P29373	Cellular retinoic acid-binding protein 2	0.012	0.004	0.004	0.002	0.003	0.003	0.113	0.017	0.003	0.010	0.004	0.008
Other Plasma components	P31151 Q86SG5	Protein S100-A7	0.010	0.016	0.016	0.023	0.025	0.025	0.047	0.023	0.012	0.018	0.017	0.029
Other Plasma components	O14544	Suppressor of cytokine signaling 6	0.032	0.097	0.094	0.049	0.044	0.058	0.064	0.054	0.066	0.044	0.073	0.052

Other Plasma components	P08567	Pleckstrin	0.061	0.082	0.086	0.105	0.081	0.112	0.063	0.118	0.077	0.098	0.082	0.070
Other Plasma components	Q8N699	Myc target protein 1	0.100	0.015	0.012	0.038	0.062	0.021	0.250	0.022	0.008	0.034	0.027	0.070
Other Plasma components	Q8N108	Mesoderm induction early response protein 1	0.008	0.014	0.008	0.019	0.021	0.015	0.007	0.055	0.023	0.036	0.117	0.020
Other Plasma components	P35858	Insulin-like growth factor-binding protein complex acid labile subunit	0.099	0.100	0.084	0.096	0.067	0.054	0.394	0.160	0.189	0.149	0.121	0.146
Other Plasma components	P10075	Zinc finger protein GLI4	0.012	0.081	0.093	0.099	0.058	0.075	0.014	0.116	0.132	0.061	0.066	0.077
Other Plasma components	Q9NQ30	Endothelial cell-specific molecule 1	0.000	0.028	0.030	0.000	0.003	0.006	0.011	0.001	0.005	0.005	0.017	0.006
Other Plasma components	P60174	Triosephosphate isomerase	0.031	0.016	0.016	0.041	0.039	0.037	0.018	0.056	0.021	0.039	0.047	0.047
Other Plasma components	P01344	Insulin-like growth factor II	0.022	0.004	0.003	0.003	0.007	0.005	0.010	0.000	0.004	0.000	0.000	0.002
Other Plasma components	P24593	Insulin-like growth factor-binding protein 5	0.005	0.021	0.021	0.008	0.007	0.009	0.004	0.001	0.008	0.002	0.001	0.002
Other Plasma components	P02765	Alpha-2-HS-glycoprotein	0.428	0.057	0.054	0.103	0.169	0.081	0.524	0.047	0.056	0.109	0.087	0.232
Other Plasma components	P05109	Protein S100-A8	0.150	0.039	0.044	0.047	0.051	0.042	0.140	0.036	0.041	0.044	0.041	0.055
Other Plasma components	P05089	Arginase-1	0.003	0.011	0.010	0.010	0.014	0.016	0.012	0.003	0.006	0.005	0.013	0.012
Other Plasma components	Q00889	Pregnancy-specific beta-1-glycoprotein 6	2.905	0.131	0.125	0.100	0.391	0.159	1.534	0.256	0.066	0.406	0.249	0.535

Other Plasma components	P31947 P61981 P62258 Q04917	14-3-3 protein sigma	0.000	0.001	0.000	0.002	0.000	0.003	0.003	0.003	0.002	0.003	0.003	0.002
Other Plasma components	P28715	DNA repair protein complementing XP-G cells	0.000	0.000	0.001	0.000	0.000	0.000	0.000	0.000	0.000	0.000	0.000	0.000
Other Plasma components	P31949	Protein S100-A11	0.000	0.001	0.001	0.001	0.002	0.001	0.002	0.001	0.001	0.003	0.000	0.001
Other Plasma components	P31267	Homeobox protein Hox-A6	0.097	0.037	0.040	0.041	0.061	0.059	0.092	0.040	0.042	0.035	0.048	0.055
Other Plasma components	Q5TCS8	Adenylate kinase 9	1.592	1.273	1.185	1.150	1.101	0.774	2.159	0.967	1.302	1.050	0.905	1.226
Other Plasma components	P15311	Ezrin	0.005	0.001	0.000	0.000	0.001	0.001	0.009	0.001	0.000	0.005	0.002	0.001
Other Plasma components	Q9BXN1	Asporin	0.000	0.001	0.001	0.000	0.000	0.000	0.006	0.003	0.002	0.002	0.003	0.002
Other Plasma components	Q8TDM0	Breast carcinoma-amplified sequence 4	0.001	0.017	0.020	0.000	0.000	0.000	0.004	0.000	0.004	0.000	0.000	0.000
Other Plasma components	Q14624	Inter-alpha-trypsin inhibitor heavy chain H4	0.112	0.317	0.266	0.209	0.215	0.165	0.097	0.106	0.068	0.093	0.092	0.090
Other Plasma components	P14136	Glial fibrillary acidic protein	0.423	0.039	0.032	0.034	0.064	0.027	0.256	0.056	0.023	0.064	0.028	0.065
Other Plasma components	P29508	Serpin B3	0.082	0.038	0.043	0.032	0.064	0.058	0.095	0.094	0.026	0.079	0.043	0.058
Other Plasma components	P32119	Peroxiredoxin-2	0.001	0.000	0.001	0.006	0.007	0.007	0.012	0.002	0.000	0.002	0.007	0.009
Other Plasma components	P07996 P49746	Thrombospondin-1	0.106	0.142	0.137	0.115	0.084	0.097	0.076	0.053	0.102	0.049	0.044	0.044

Other Plasma components	P80723	Brain acid soluble protein 1	1.877	0.164	0.182	0.258	0.413	0.276	1.295	0.218	0.196	0.318	0.236	0.388
Other Plasma components	P10720	Platelet factor 4 variant	0.003	0.001	0.001	0.001	0.001	0.001	0.004	0.004	0.024	0.003	0.005	0.006
Other Plasma components	Q7Z4G4	tRNA (guanine(10)-N2)-methyltransferase homolog	0.008	0.009	0.008	0.004	0.004	0.003	0.040	0.002	0.005	0.003	0.003	0.002
Other Plasma components	P60484	Phosphatidylinositol 3,4,5-trisphosphate 3-phosphatase and dual-specificity protein phosphatase PTEN	0.156	0.018	0.015	0.023	0.017	0.012	0.203	0.019	0.042	0.031	0.031	0.027
Other Plasma components	P51888	Prolargin	0.003	0.006	0.006	0.005	0.003	0.004	0.009	0.017	0.006	0.011	0.006	0.005
Other Plasma components	P13284	Gamma-interferon-inducible lysosomal thiol reductase	1.649	0.154	0.168	0.193	0.369	0.163	1.058	0.132	0.086	0.220	0.112	0.290
Other Plasma components	P02042	Hemoglobin subunit delta	0.002	0.003	0.005	0.001	0.001	0.001	0.002	0.000	0.000	0.000	0.001	0.001
Other Plasma components	P69905	Hemoglobin subunit alpha	0.038	0.027	0.030	0.034	0.036	0.034	0.079	0.026	0.015	0.024	0.031	0.031
Other Plasma components	P20742	Pregnancy zone protein	0.025	0.051	0.056	0.053	0.040	0.057	0.069	0.061	0.087	0.041	0.038	0.040
Other Plasma components	P80188	Neutrophil gelatinase-associated lipocalin	0.001	0.001	0.001	0.001	0.000	0.000	0.005	0.000	0.000	0.000	0.000	0.000
Other Plasma components	Q7L273	BTB/POZ domain-containing protein KCTD9	1.158	0.165	0.146	0.115	0.124	0.082	0.271	0.143	0.197	0.198	0.183	0.280

Other Plasma components	Q15828	Cystatin-M	0.013	0.034	0.030	0.028	0.018	0.020	0.019	0.022	0.045	0.020	0.019	0.016
Other Plasma components	O75223	Gamma-glutamylcyclotransferase	0.029	0.019	0.016	0.014	0.024	0.020	0.008	0.026	0.013	0.026	0.035	0.027
Other Plasma components	P34931	Heat shock 70 kDa protein 1-like	4.493	0.474	0.494	0.715	1.067	0.540	2.633	0.459	0.432	0.820	0.398	1.119
Other Plasma components	P36952	Serpin B5	0.002	0.000	0.000	0.000	0.000	0.000	0.010	0.000	0.000	0.000	0.001	0.001
Other Plasma components	Q9NYJ1	Cytochrome c oxidase assembly factor 4 homolog, mitochondrial	0.165	0.140	0.151	0.099	0.090	0.075	0.206	0.039	0.073	0.050	0.050	0.049
Other Plasma components	Q5T197	DC-STAMP domain-containing protein 1	0.023	0.023	0.027	0.015	0.013	0.012	0.089	0.007	0.034	0.008	0.010	0.015
Other Plasma components	P15924	Desmoplakin	0.025	0.001	0.001	0.001	0.001	0.001	0.009	0.002	0.003	0.001	0.001	0.002
Other Plasma components	Q9UK55	Protein Z-dependent protease inhibitor	0.001	0.024	0.007	0.004	0.002	0.003	0.073	0.005	0.008	0.015	0.003	0.009
Other Plasma components	Q14116	Interleukin-18	1.010	0.082	0.099	0.142	0.182	0.094	0.362	0.116	0.081	0.105	0.072	0.210
Other Plasma components	O43278	Kunitz-type protease inhibitor 1	0.027	0.023	0.023	0.016	0.024	0.025	0.030	0.017	0.015	0.025	0.012	0.019
Other Plasma components	Q9BXU0	Testis-expressed protein 12	0.000	0.000	0.000	0.000	0.000	0.000	0.000	0.000	0.000	0.000	0.000	0.000
Other Plasma components	P19971	Thymidine phosphorylase	0.000	0.000	0.000	0.000	0.000	0.000	0.003	0.000	0.000	0.000	0.000	0.000
Other Plasma components	Q5JR12	Protein phosphatase 1J	0.000	0.000	0.000	0.000	0.000	0.000	0.033	0.000	0.000	0.000	0.000	0.000
Other Plasma components	Q8N5X7	Eukaryotic translation initiation factor 4E type 3	0.092	0.049	0.048	0.056	0.051	0.038	0.114	0.052	0.119	0.058	0.048	0.064

Other Plasma components	Q9NTU7	Cerebellin-4	0.001	0.003	0.003	0.012	0.011	0.018	0.001	0.011	0.008	0.017	0.013	0.012
Other Plasma components	P23528 Q9Y281	Cofilin-1	0.038	0.024	0.025	0.020	0.024	0.018	0.023	0.006	0.010	0.009	0.011	0.019
Other Plasma components	P57768	Sorting nexin-16	0.000	0.000	0.000	0.000	0.000	0.000	0.009	0.000	0.000	0.000	0.000	0.000
Other Plasma components	P04792	Heat shock protein beta-1	0.018	0.009	0.007	0.009	0.006	0.008	0.029	0.051	0.194	0.050	0.076	0.064
Other Plasma components	Q9NY97	N-acetyllactosaminide beta-1,3-N-acetylglucosaminyltransferase 2	0.000	0.000	0.000	0.000	0.000	0.000	0.000	0.000	0.000	0.000	0.000	0.000
Other Plasma components	P01040	Cystatin-A	0.000	0.001	0.000	0.000	0.001	0.001	0.009	0.000	0.001	0.001	0.000	0.000
Other Plasma components	P02760	Protein AMBP	0.049	0.028	0.027	0.036	0.022	0.030	0.083	0.043	0.049	0.046	0.043	0.042
Other Plasma components	P58550	Putative FXYD domain-containing ion transport regulator 8	0.021	0.030	0.036	0.049	0.030	0.051	1.715	0.030	0.055	0.041	0.035	0.043
Other Plasma components	Q2TV78	Putative macrophage stimulating 1-like protein	0.028	0.028	0.030	0.025	0.027	0.025	0.081	0.046	0.027	0.031	0.032	0.040
Other Plasma components	A8K2U0	Alpha-2-macroglobulin-like protein 1	0.010	0.035	0.029	0.015	0.007	0.014	0.107	0.046	0.051	0.048	0.038	0.041
Other Plasma components	Q5TAB7	Protein ripply2	0.019	0.049	0.052	0.028	0.037	0.049	0.008	0.010	0.014	0.001	0.006	0.004
Other Plasma components	Q9UL33	Trafficking protein particle complex subunit 2-like protein	0.101	0.076	0.070	0.064	0.074	0.068	0.065	0.088	0.114	0.137	0.203	0.215

Other Plasma components	P61278	Somatostatin	0.000	0.001	0.001	0.003	0.001	0.001	0.000	0.003	0.000	0.001	0.000	0.001
Other Plasma components	Q14CZ7	FAST kinase domain-containing protein 3, mitochondrial	0.100	0.098	0.100	0.064	0.049	0.055	0.149	0.051	0.075	0.051	0.039	0.050
Other Plasma components	P0CG38	POTE ankyrin domain family member I	0.054	0.157	0.143	0.163	0.102	0.121	0.085	0.029	0.040	0.015	0.016	0.017
Other Plasma components	P01236	Prolactin	0.050	0.069	0.066	0.059	0.042	0.051	0.106	0.001	0.000	0.000	0.002	0.002
Other Plasma components	P02766	Transthyretin	0.319	0.135	0.127	0.070	0.103	0.112	0.304	0.190	0.141	0.219	0.238	0.167
Other Plasma components	P00338	L-lactate dehydrogenase A chain	0.022	0.011	0.015	0.014	0.014	0.016	0.029	0.005	0.006	0.007	0.007	0.010
Other Plasma components	P47929	Galectin-7	0.015	0.036	0.034	0.030	0.026	0.027	0.211	0.037	0.040	0.035	0.031	0.027
Other Plasma components	P19823	Inter-alpha-trypsin inhibitor heavy chain H2	0.216	0.053	0.057	0.077	0.074	0.103	0.303	0.222	0.430	0.287	0.434	0.305
Other Plasma components	P31944	Caspase-14	0.023	0.018	0.017	0.017	0.020	0.016	0.049	0.033	0.016	0.018	0.026	0.022
Other Plasma components	Q6ZRG5	Putative uncharacterized protein FLJ43944	0.005	0.004	0.005	0.005	0.007	0.006	0.029	0.010	0.009	0.008	0.009	0.011
Other Plasma components	Q14244	Ensconsin	0.017	0.015	0.012	0.013	0.005	0.010	0.008	0.000	0.001	0.000	0.000	0.001
Tissue Leakage	P05452	Tetranectin	0.054	0.060	0.060	0.025	0.023	0.023	0.123	0.025	0.083	0.022	0.027	0.031
Tissue Leakage	P35579	Myosin-9	0.219	0.017	0.020	0.021	0.039	0.016	0.873	0.014	0.012	0.018	0.019	0.044
Tissue Leakage	P62736 P63267 P68032 P68133	Actin, aortic smooth muscle	0.497	0.047	0.049	0.101	0.147	0.065	0.705	0.060	0.049	0.103	0.069	0.142

Tissue Leakage	P35908	Keratin, type II cytoskeletal 2 epidermal	0.062	0.414	0.467	0.333	0.277	0.347	0.141	0.583	0.423	0.429	0.372	0.334
Tissue Leakage	P06396	Gelsolin	0.029	0.506	0.481	0.210	0.168	0.148	0.060	0.094	0.422	0.155	0.116	0.166
Tissue Leakage	P26038	Moesin	0.022	0.682	0.656	0.835	0.813	0.714	0.137	0.396	0.495	0.501	0.345	0.377
Tissue Leakage	P13645<br>Q7Z3Y7<br>Q7Z3Y8<br>Q7Z3Z0	Keratin, type I cytoskeletal 10	0.041	0.216	0.157	0.167	0.129	0.188	0.057	0.179	0.202	0.174	0.143	0.131
Tissue Leakage	P02533	Keratin, type I cytoskeletal 14	0.064	0.012	0.008	0.006	0.011	0.008	0.062	0.007	0.008	0.012	0.010	0.008
Tissue Leakage	P04259<br>P12035<br>P12036<br>Q01546	Keratin, type II cytoskeletal 6B	0.088	0.211	0.208	0.278	0.307	0.358	0.135	0.424	0.296	0.345	0.431	0.354
Tissue Leakage	P63261<br>P60709<br>Q562R1<br>Q9BYX7	Actin, cytoplasmic 2	0.055	0.140	0.164	0.130	0.098	0.120	0.062	0.140	0.138	0.151	0.094	0.083
Tissue Leakage	P04350<br>P07437<br>P68371	Tubulin beta-4A chain	0.014	0.530	0.479	0.087	0.101	0.174	0.016	0.108	0.449	0.135	0.256	0.097
Tissue Leakage	Q96PD5	N-acetylmuramoyl-L-alanine amidase	0.114	0.060	0.059	0.069	0.123	0.055	0.128	0.079	0.046	0.094	0.057	0.156
Tissue Leakage	P15169	Carboxypeptidase N catalytic chain	0.404	0.264	0.241	0.309	0.329	0.286	0.329	0.273	0.181	0.263	0.303	0.292
Tissue Leakage	Q71U36<br>P68363<br>P68366<br>Q13748	Tubulin alpha-1A chain	0.020	0.041	0.035	0.042	0.048	0.054	0.028	0.127	0.034	0.081	0.094	0.109

	Q6PEY2<br>Q9BQE3<br>Q9NY65													
Tissue Leakage	P04004	Vitronectin	0.299	5.373	5.119	1.046	1.159	2.203	0.235	0.821	3.293	1.213	2.625	1.172
Tissue Leakage	P06702	Protein S100-A9	0.018	0.027	0.035	0.030	0.027	0.031	0.139	0.033	0.033	0.034	0.042	0.032
Tissue Leakage	P03950	Angiogenin	0.067	0.105	0.116	0.096	0.091	0.103	0.194	0.126	0.116	0.103	0.119	0.104
Tissue Leakage	Q04695<br>O76014<br>P08727<br>Q14525<br>Q99456	Keratin, type I cytoskeletal 17	0.036	0.027	0.028	0.023	0.018	0.019	0.058	0.019	0.018	0.016	0.014	0.019
Tissue Leakage	P62937	Peptidyl-prolyl cis-trans isomerase A	0.032	0.017	0.016	0.015	0.018	0.020	0.010	0.034	0.023	0.031	0.024	0.024
Tissue Leakage	P02538	Keratin, type II cytoskeletal 6A	0.023	0.052	0.048	0.069	0.083	0.095	0.038	0.112	0.050	0.091	0.108	0.089
Tissue Leakage	P48668<br>O95678	Keratin, type II cytoskeletal 6C	0.016	0.037	0.034	0.049	0.059	0.068	0.027	0.080	0.036	0.064	0.077	0.063
Tissue Leakage	Q9C075	Keratin, type I cytoskeletal 23	0.006	0.007	0.009	0.004	0.004	0.004	0.005	0.006	0.005	0.005	0.005	0.004
Tissue Leakage	Q92954	Proteoglycan 4	0.033	0.786	0.753	0.091	0.064	0.046	0.101	0.117	0.509	0.142	0.100	0.136
Tissue Leakage	Q5T749	Keratinocyte proline-rich protein	0.000	0.001	0.000	0.000	0.000	0.000	0.010	0.001	0.001	0.000	0.000	0.000
Tissue Leakage	P05787	Keratin, type II cytoskeletal 8	0.005	0.009	0.008	0.016	0.022	0.025	0.007	0.029	0.009	0.021	0.029	0.024
Tissue Leakage	Q8N1N4	Keratin, type II cytoskeletal 78	0.001	0.000	0.000	0.001	0.001	0.000	0.000	0.001	0.000	0.001	0.001	0.001
Tissue Leakage	P04264	Keratin, type II cytoskeletal 1	0.106	0.175	0.070	0.080	0.061	0.060	0.163	0.070	0.087	0.078	0.074	0.100

Tissue Leakage	Q04756	Hepatocyte growth factor activator	0.019	0.205	0.209	0.060	0.063	0.046	0.186	0.047	0.145	0.065	0.040	0.041
Tissue Leakage	Q7Z3Y9	Keratin, type I cytoskeletal 26	0.011	0.008	0.011	0.012	0.020	0.025	0.013	0.043	0.007	0.033	0.015	0.024
Tissue Leakage	A5A3E0	POTE ankyrin domain family member F	0.003	0.002	0.002	0.001	0.000	0.000	0.013	0.002	0.002	0.001	0.000	0.000
Tissue Leakage	P62805	Histone H4	0.005	0.004	0.004	0.002	0.002	0.002	0.006	0.002	0.003	0.004	0.002	0.002
Tissue Leakage	P02788	Lactotransferrin	0.121	0.068	0.070	0.058	0.036	0.050	0.105	0.063	0.099	0.050	0.041	0.044
Tissue Leakage	P07737	Profilin-1	0.000	0.000	0.000	0.000	0.000	0.000	0.003	0.000	0.000	0.000	0.000	0.000
Tissue Leakage	P63104	14-3-3 protein zeta/delta	0.006	0.001	0.001	0.001	0.002	0.001	0.012	0.002	0.001	0.003	0.001	0.004
Tissue Leakage	P08779	Keratin, type I cytoskeletal 16	0.039	0.142	0.089	0.090	0.060	0.064	0.045	0.055	0.137	0.069	0.078	0.065
Tissue Leakage	P06733	Alpha-enolase	0.085	0.096	0.086	0.084	0.103	0.094	0.083	0.243	0.095	0.231	0.173	0.119
Tissue Leakage	P30043	Flavin reductase (NADPH)	0.010	0.010	0.011	0.009	0.010	0.008	0.019	0.008	0.008	0.008	0.007	0.006
Tissue Leakage	O00391	Sulfhydryl oxidase 1	0.033	0.049	0.048	0.029	0.031	0.032	0.142	0.095	0.115	0.085	0.125	0.105
Tissue Leakage	P19012	Keratin, type I cytoskeletal 15	0.193	0.032	0.035	0.049	0.081	0.063	0.143	0.064	0.037	0.057	0.056	0.070
Tissue Leakage	P14618	Pyruvate kinase PKM	0.002	0.001	0.001	0.002	0.001	0.002	0.011	0.003	0.002	0.002	0.002	0.001
Tissue Leakage	P13647	Keratin, type II cytoskeletal 5	0.046	0.013	0.015	0.017	0.018	0.017	0.091	0.034	0.029	0.028	0.037	0.033
Tissue Leakage	P08729	Keratin, type II cytoskeletal 7	0.175	0.047	0.042	0.037	0.033	0.018	0.309	0.051	0.054	0.062	0.054	0.055
Tissue Leakage	Q9Y4G6	Talin-2	0.214	0.170	0.146	0.128	0.190	0.146	0.159	0.218	0.124	0.249	0.292	0.218
Tissue Leakage	P35527	Keratin, type I cytoskeletal 9	0.005	0.089	0.006	0.028	0.002	0.004	0.035	0.007	0.026	0.010	0.014	0.028
Tissue Leakage	Q86UX7	Fermitin family homolog 3	0.020	0.031	0.031	0.032	0.033	0.035	0.050	0.026	0.014	0.029	0.027	0.022

Tissue Leakage	Q15113	Procollagen C-endopeptidase enhancer 1	0.126	0.098	0.111	0.065	0.089	0.090	0.058	0.093	0.102	0.087	0.068	0.069
Tissue Leakage	O43866	CD5 antigen-like	0.061	0.047	0.051	0.044	0.048	0.048	0.112	0.052	0.039	0.061	0.067	0.068
Tissue Leakage	Q7Z794	Keratin, type II cytoskeletal 1b	0.311	0.051	0.050	0.077	0.085	0.058	0.144	0.101	0.047	0.084	0.086	0.111
Tissue Leakage	Q9Y490	Talin-1	0.337	0.117	0.114	0.055	0.056	0.063	0.209	0.061	0.102	0.067	0.146	0.083
Tissue Leakage	Q15323	Keratin, type I cuticular Ha1	0.420	0.080	0.067	0.079	0.120	0.089	0.420	0.250	0.207	0.240	0.322	0.270
Tissue Leakage	P04406	Glyceraldehyde-3-phosphate dehydrogenase	0.043	0.048	0.040	0.053	0.055	0.058	0.138	0.006	0.008	0.005	0.010	0.009
Tissue Leakage	P47755	F-actin-capping protein subunit alpha-2	0.037	0.041	0.038	0.053	0.033	0.043	0.125	0.005	0.005	0.006	0.008	0.007
Tissue Leakage	P27105	Erythrocyte band 7 integral membrane protein	0.000	0.000	0.000	0.000	0.000	0.000	0.005	0.000	0.000	0.000	0.002	0.001
Tissue Leakage	P04075	Fructose-bisphosphate aldolase A	0.001	0.013	0.010	0.003	0.005	0.002	0.040	0.000	0.004	0.001	0.004	0.001
Tissue Leakage	Q5XKE5	Keratin, type II cytoskeletal 79	0.073	0.090	0.096	0.075	0.077	0.082	0.391	0.077	0.068	0.056	0.058	0.063
Tissue Leakage	Q92764	Keratin, type I cuticular Ha5	0.149	0.013	0.013	0.012	0.008	0.003	0.188	0.034	0.050	0.060	0.078	0.052
